# Research Progress on the Immunomodulatory Effects by Dang Gui (*Radix Angelica sinensis*) and Its Active Chemical Components

**DOI:** 10.3390/molecules31071153

**Published:** 2026-03-31

**Authors:** Tong Li, Xueying Zhao

**Affiliations:** School of Basic Medical Sciences, Heilongjiang University of Chinese Medicine, 24 Heping Road, Harbin 150040, China; litong199809@163.com

**Keywords:** Dang Gui, *Radix Angelica sinensis*, active chemical components, regulation of immune response

## Abstract

Dang Gui (*Radix Angelica sinensis*), a classic Chinese medicinal herb, is renowned for nourishing blood, promoting blood circulation, regulating meridians, and relieving pain, and widely used clinically for anemia, cancer, rheumatoid arthritis, ulcerative colitis, and other diseases. Studies have confirmed that Dang Gui and its major bioactive components (e.g., polysaccharides, ferulic acid, (Z)-ligustilide) exert diverse pharmacological activities including immunomodulation, neuroprotection, and hepatoprotection. Based on a systematic literature search, this review summarizes the traditional applications and main chemical constituents of *A. sinensis*, with a focused analysis of its immunomodulatory effects. Evidence shows that *A. sinensis* exerts bidirectional immunoregulation by improving immune organ indices, promoting the proliferation and activation of immune cells, including T/B lymphocytes (T/B cells), macrophages, and regulating cytokine secretion. Furthermore, it reviews its immunomodulatory mechanisms in immune-related diseases (e.g., cancer, aplastic anemia, chronic pain), analyzes its quality control standards from regulatory and pharmacopeial perspectives and summarizes relevant safety research. This review comprehensively integrates the immunoregulatory effects and underlying mechanisms of *A. sinensis*, aiming to provide a scientific basis for its future research and clinical application.

## 1. Introduction

*Angelica sinensis* (Oliv.) Diels (*A. sinensis*), a member of the Apiaceae family (formerly Umbelliferae), is commonly known as “Dang Gui” or “female ginseng”. First recorded in the *Shennong’s Classic of Materia Medica*, this medium-grade herb has a pungent–sweet flavor, warm nature, and belongs to the heart, liver, and spleen meridians, with core functions of promoting blood circulation, regulating meridians, relieving pain, and moistening the intestines. Classic traditional Chinese medicine (TCM) formulas containing *A. sinensis* (e.g., Dang Gui Shao Yao San, Dang Gui Si Ni Decoction) have long been clinically applied, and relevant studies have confirmed their efficacy and safety in treating primary dysmenorrhea [1], diabetic peripheral neuropathy [2], menopause symptoms [3], and hyperthyroidism [4].

Recent studies have revealed that *A. sinensis* and its compound formulas possess complex chemical compositions, including terpenoids, phenolic acids, volatile oils, and polysaccharides [5]. These constituents contribute to a range of pharmacological activities, including immunomodulation and antioxidation [6,7]. Mu et al. [8] found that *Angelica sinensis* polysaccharides (ASP) can prevent D-galactose induced hematopoietic stem cell aging and reduce oxidative damage. Zhang et al. [9] demonstrated that ASP can ameliorates iron deficiency anemia caused by hepcidin overexpression through inhibition of hepcidin expression and modulation of iron metabolism. Lang et al. [10] revealed that (Z)-ligustilide induces oxidative stress, thereby promoting apoptosis in ovarian cancer cells.

Existing reviews on *A. sinensis* are limited by their narrow focus—either focusing on a single pharmacological effect (e.g., anti-tumor or hematopoietic activity) or individual active components, failing to systematically summarize its immunomodulatory mechanisms across multiple biological levels. More importantly, they lack integration of pharmacopeial regulations, quality control standards, and safety research, which are critical for clinical application and industrialization. It systematically integrates research on the regulation of immune organs, immune cells, and immune-active substances by *A. sinensis* and its key components (e.g., ASP, ferulic acid, (Z)-ligustilide); further elaborates on their immunomodulatory roles in immune-mediated diseases; and supplements discussions on pharmacopeial compliance, quality control, and safety. Therefore, this comprehensive perspective fills the gaps in existing literature, offering more systematic and practical insights for the clinical application and in-depth mechanistic research of *A. sinensis*.

## 2. Literature Search Strategy

A systematic literature search was conducted per a prespecified strategy to ensure a comprehensive, reproducible review of the immunomodulatory effects of *A. sinensis* and its constituents.

### 2.1. Databases and Time Frame

Electronic searches were performed in three key databases: PubMed, Web of Science (Core Collection), and China National Knowledge Infrastructure (CNKI). The search covered all literature from each database’s inception to December 2025, encompassing both foundational and the latest relevant studies.

### 2.2. Search Terms

A combination of database-specific subject headings (e.g., Medical Subject Headings (MeSH) in PubMed) and free-text keywords was used. Core terms included: *Angelica sinensis*, Dang Gui, *Radix Angelica sinensis*, *Angelica sinensis* polysaccharide, polysaccharide AND *Radix Angelica sinensis*, ligustilide, ferulic acid, immunomodulate, immune response, immune system, cytokine, macrophage, lymphocyte, thymus, spleen. Additional terms for quality control and standardization were incorporated: pharmacopeia, quality control, standardization, marker compound, processing, and herbal medicine standardization. Boolean operators (AND, OR) were used to combine terms for capturing studies on chemical constituents, quality standards, immune-related outcomes, and disease models.

### 2.3. Inclusion and Exclusion Criteria

Studies were screened by the following criteria: Including (1) original in vitro, in vivo or clinical research, or relevant reviews focusing on *A. sinensis* (whole extract, formulations, isolated constituents); (2) studies reporting quantifiable immune function outcomes (e.g., immune organ indices, immune cell activity, cytokine levels) or immune-mediated diseases (e.g., cancer, anemia, arthritis); (3) English or Chinese publications. Excluding: (1) duplicate publications; (2) non-peer-reviewed conference abstracts, editorials, letters or unpublished theses; (3) studies with inadequate methodological description or missing ethical approval (animal/human studies); (4) articles unrelated to the immunomodulatory effects of *A. sinensis*.

## 3. Traditional Uses

The medicinal history of *A. sinensis* is extremely long. It was first recorded in the *Shennong’s Classic of Materia Medica* and classified as a medium-grade herb. Over time, medical practitioners have continuously enriched our understanding of its efficacy and applications, establishing comprehensive compatibility theories and numerous classic formulas, including Dang Gui Si Ni Decoction, Dang Gui Shao Yao San, and Dang Gui Bu Xue Decoction. These preparations remain widely used in clinical practice to date. Clinical observations have demonstrated that compounds mainly consisting of *A. sinensis* exhibit promising therapeutic potential against cancer, anemia, osteoarthritis, and neurodegenerative diseases, primarily by alleviating clinical symptoms, regulating immune function, and improving the quality of life of patients. Furthermore, they also show favorable application prospects in gynecological disorders, digestive system diseases, and diabetic complications. The traditional and modern clinical applications of *A. sinensis* are summarized in Table 1.

Based on the above discussion, Dang Gui is widely used in clinical traditional Chinese medicine, often in combination with other herbs to treat a diverse range of conditions, including cancer, anemia, osteoarthritis, neurodegenerative diseases, gynecological disorders (e.g., dysmenorrhea), and diabetic nephropathy. Clinical evidence supports its favorable efficacy and safety profile. Notably, studies have demonstrated that *A. sinensis*-containing formulations can modulate human immune function and suppress the expression of inflammatory factors, thereby contributing to their anti-inflammatory and therapeutic effects.

However, the chemical composition of *A. sinensis*-based compound prescriptions is highly complex. Formulations such as Dang Gui Bu Xue Decoction and Dang Gui Shao Yao San have been shown to contain multiple bioactive constituents, including polysaccharides, volatile oils, and organic acids. Given the multi-target and multi-pathway characteristics of traditional Chinese medicine, a critical challenge is to deconvolute this complexity and identify the specific components responsible for the observed immunological effects. Addressing this issue requires systematic chemical and pharmacological analyses to clarify the active constituents of *A. sinensis* and their specific impacts on the immune system. Such investigations will facilitate a deeper understanding of the immunomodulatory mechanisms of its key components in diseases including cancer, anemia, and osteoarthritis, ultimately providing a scientific basis for future research on *A. sinensis*.

## 4. Chemical Constituents of *Angelica sinensis*

### 4.1. Angelica sinensis Polysaccharides

*Angelica sinensis* polysaccharides (ASP), water-soluble polysaccharides isolated from *A. sinensis*, represent one of its major bioactive constituents. They are mainly composed of monosaccharide units (e.g., galactose, mannose, glucose, and rhamnose) linked by α- or β-glycosidic bonds. Cao et al. [29] extracted two arabinogalactan fractions, designated APS-1cI and APS-1cII. APS-1cI was a linear α-glucan composed exclusively of (1→6)-α-D-Glcp, while APS-1cII had a repeating unit consisting of (1→4)-α-D-Glcp and (1→6)-α-D-Glcp at a molar ratio of 4:1. Another study reported that polysaccharides extracted from *A. sinensis* roots consist of rhamnose, galacturonic acid, glucose, galactose, and arabinose at a molar ratio of 0.05:0.26:14.47:1.00:1.17 [30]. Zhao et al. [31] isolated two polysaccharide fractions, APS-1a and APS-3a, from *A. sinensis* and performed structural characterization. APS-1a was predominantly composed of glucose, with galactose and a small amount of arabinose. Its core structure was a linear chain of 1,4-linked β-D-Glcp, interspersed with a small quantity of 1,4-linked β-D-Galp, forming a mixed main chain of “Glc-Glc-Gal-Glc-…”. APS-3a contained glucose, galactose, glucuronic acid, and a small amount of arabinose. Its structural core was a linear chain of 1,4-linked β-D-GalAp (showing acidic characteristics), interspersed with 1,4-linked β-D-Glcp, forming an acidic main chain of “GalA-GalA-Glc-GalA-…”. Additionally, Wang et al. [32] obtained four distinct polysaccharide fractions from *A. sinensis* and elucidated their structures. Their results revealed that all four polysaccharides shared a linear backbone formed by D-pyranose residues connected via 1,4-glycosidic linkages, although slight variations existed in their core structural motifs.

Additionally, ASP can exert a variety of immunomodulatory effects. And the structural complexity of ASP and the structural differences caused by different extraction methods, further studies on their structure–activity relationship are still required. Commonly used extraction methods for ASP include hot-water extraction, ethanol precipitation, and ultrasonic-assisted extraction. The choice of extraction method significantly affects both the extraction yield and the bioactivity of the obtained polysaccharides. Conventional hot-water extraction generally requires long-term heating at high temperatures, which not only results in relatively low extraction efficiency but may also cause polysaccharide degradation and reduced biological activity [33]. In a comparative study, Nai et al. [34] reported that ASP could be obtained by both hot-water extraction and ultrasonic extraction, with the latter showing higher extraction efficiency.

### 4.2. Phthalein Compounds

**Figure 1:**
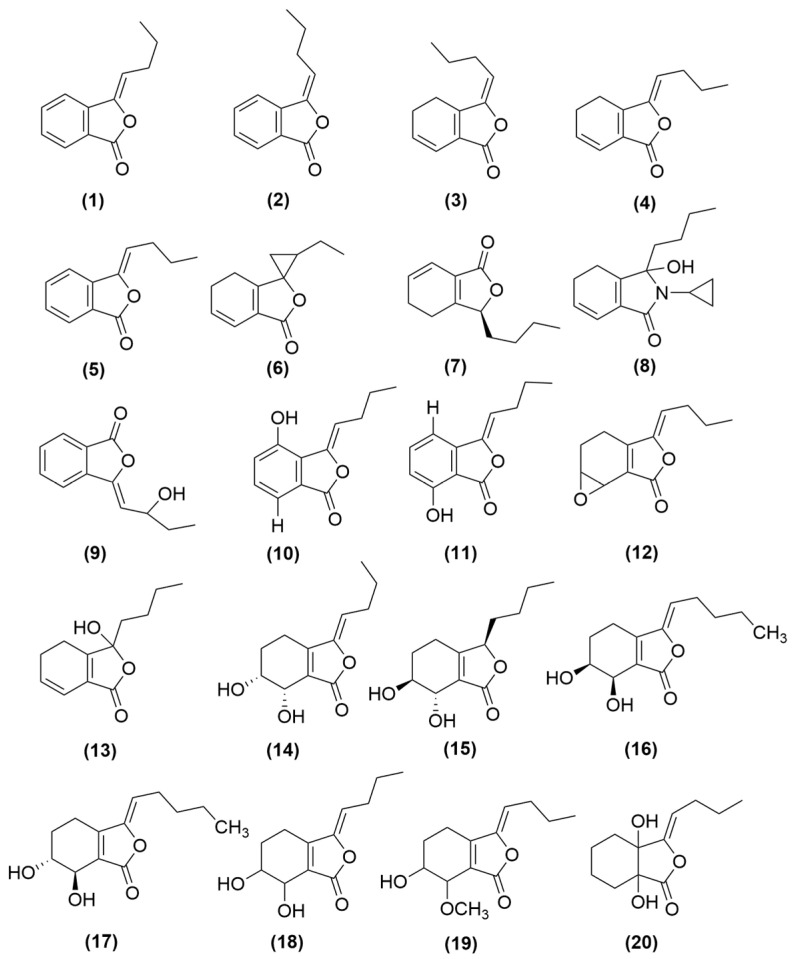
Phthalide monomers **1**–**20** isolated from Dang Gui.

**Figure 2:**
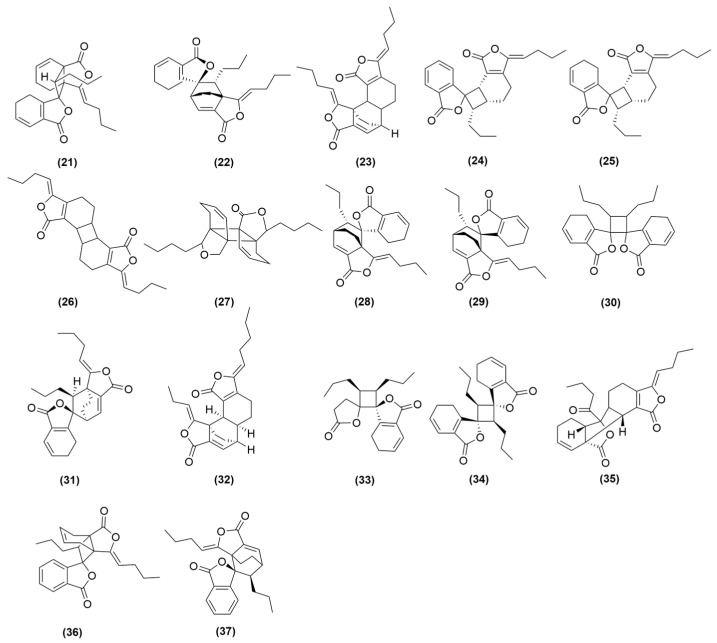
Phthalein dimers **21**–**37** isolated from Dang Gui.

Phthalein compounds include phthalein monomers (**1**–**20**) and phthalein dimers (**21**–**37**) in *A. sinensis* volatile oil, all of which are illustrated in Figure 1 and Figure 2. Phthalide monomers include Z-butylidenephthalide (**1**), E-butylidenephthalide (**2**), (Z)-ligustilide (**3**), (E)-ligustilide (**4**), n-butylphthalide (**5**), neoligustilide (**6**), senkyunolide A (**7**), ligusticum cycloprolactam (LIGc) (**8**), senkyunolide F (**9**), 3-butylidene-4-hydroxyphthalide (**10**), 3-butylidene-7-hydroxyphthalide (**11**), Z-6,7-epoxyligustilide (**12**), senkyunolide G (**13**), senkyunolide H (**14**), senkyunolide J (**15**), homosenkyunolide I (**16**), homosenkyunolide H (**17**), 6,7-dihydroxyligustilide (**18**), Z-6-hydroxy-7-methoxydihydroligustilide (**19**), 3a,7a-dihydroxyligutilide (**20**). Phthalide dimers include neodiligustilide (**21**), (Z)-ligustilide dimer E-232 (**22**), senkyunolide O (**23**), gelispirolide (**24**), riligustilide (**25**), 3,3′Z-6.7′,7.6′-diligustilide (**26**), 3a.7′a,7a.3′a-diligustilide (**27**), tokinolide B (**28**), isotokinolide B (**29**), angelicolide (**30**), (3Z)-(3aR, 6S, 3′R, 8′, 8)-3a.8′,6.3-diligustilide (**31**), levistolide A (**32**), E,E-3.3′,8.8′-diligustilide (**33**), E,E′-3.8′,8.3′-isodiligustilide (**34**), Z-3′,8′,3′a,7′a-tetrahydro-6,3, 7.7′a-diligustilide-8′-one (**35**), sinaspirolide (**36**), ansaspirolide (**37**) [33,35,36,37,38].

In conclusion, phthalides are the major bioactive components of the volatile oil from *A. sinensis.* Among them, (Z)-ligustilide (**3**, Figure 1) is a major phthalide and the most abundant compound in volatile oil of *A. sinensis*. It has been demonstrated to regulate macrophage polarization, suppress the release of pro-inflammatory cytokines, and protect against thymic immune senescence. However, current research has predominantly focused on (Z)-ligustilide monomers. The immunological activities of its dimers and other derivatives remain to be systematically evaluated. Furthermore, the poor stability of phthalides may compromise the reproducibility of their pharmacological effects. Future studies should establish robust quality control methods and explore the immunological activity profiles of individual phthalides, as well as their synergistic effects.

### 4.3. Phenylpropanoids

**Figure 3:**
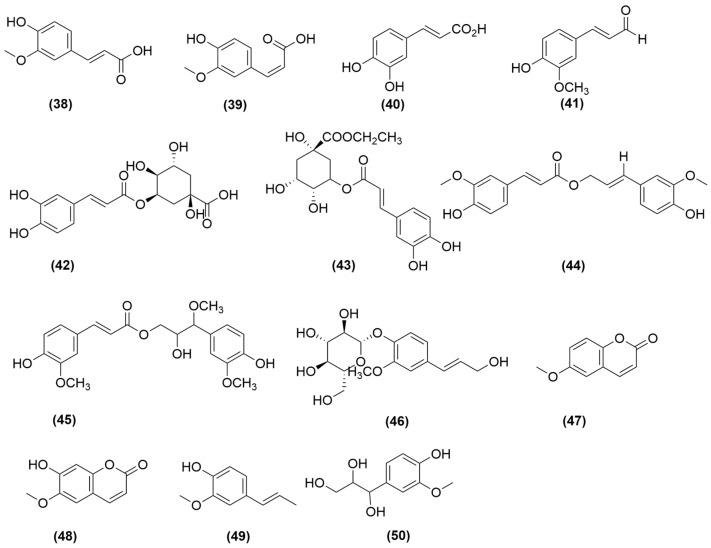
Phenylpropanoids **38**–**50** isolated from Dang Gui.

Phenylpropanoids compounds in *A. sinensis* include phenolic acids and their derivatives (**38**–**43**), phenylpropanoid esters (**44**–**45**), phenylpropanoid glycosides (**46**), coumarins (**47**–**48**), and other related compounds(**49**–**50**), including ferulic acid (**38**), cis-ferulic acid (**39**), caffeic acid (**40**), ferulic aldehyde (**41**), chlorogenic acid (**42**), 3-O-caffeoyl-D-quinic acid ethyl ester (**43**), coniferyl ferulate (**44**), angeliferulate (**45**), E-coniferin (**46**), 6-methoxy-coumarin (**47**), 6-methoxy-7-hydroxycoumarin (**48**), isoeugenol (**49**), and guaiacylglycerol (**50**) [35,37,39,40]. These phenylpropanoids are illustrated in Figure 3.

Among these, ferulic acid (**38**, Figure 3) is the most abundant [41], with the ability to regulate Th2 immune responses, suppress pro-inflammatory cytokine release and protect osteoblasts. Research has found that ferulic acid varies across different parts of *A. sinensis*, with the highest concentration found in the root tail segment [42,43]. Furthermore, Xu et al. [44] found that precipitation and temperature have an effect on the content of ferulic acid and chlorogenic acid in *A. sinensis*. Although the immunomodulatory potential of other phenolic acids, such as caffeic acid (**40**, Figure 3), chlorogenic acid (**42**, Figure 3) and coumarins has been preliminarily reported, systematic research is still lacking. Future studies should clarify their contribution to the overall immunomodulatory effects of *A. sinensis* based on the spectrum–activity relationship.

### 4.4. Terpenoids

**Figure 4:**
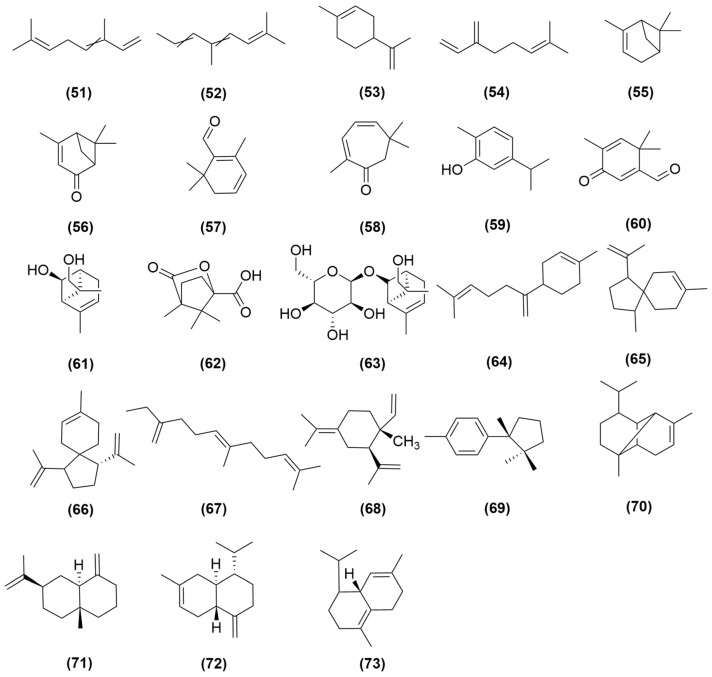
Terpenoids **51**–**73** isolated from Dang Gui.

Research has found that monoterpenoids (**51**–**63**) and sesquiterpenoids (**64**–**73**) are the components of essential oil in *A. sinensis*, including β-ocimene (**51**), allo-ocimene (**52**), limonene (**53**), myrcene (**54**), α-pinene (**55**), verbenone (**56**), safranal (**57**), eucarvone (**58**), carvacrol (**59**), 1,1,5-trimethyl-2-formyl-cyclohexa-2,5-diene-4-one (**60**), 6β,9-hydroxy-(+)-α-pinene (**61**), camphanic acid (**62**), 9-hydroxy-(+)-α-pinene-6β-O- D-glucoside (**63**)*,* β-bisabolene (**64**), isoacoradien (**65**), acoradiene (**66**), trans-β-farnesene (**67**), γ-elemene (**68**), cuparene (**69**), copaene (**70**), β-selinene (**71**), γ-cadinene (**72**), bergamotone (**73**) [45,46], all of which are illustrated in Figure 4.

The terpenoids (monoterpenes and sesquiterpenes) in *A. sinensis*, primarily identified via Finnigan Trace Mass gas chromatography-mass spectrometer (GC-MS, Thermo Fisher Scientific Inc., Waltham, MA, USA), are integral components of its volatile oil. However, immunopharmacological research on these terpenoids remains limited, and current evidence is insufficient to confirm their direct contribution to the herb’s immunomodulatory effects. Given their low abundance and high structural diversity, future studies should adopt activity-guided fractionation strategies combined with in vitro immune screening to identify key bioactive components from the complex mixture and elucidate their underlying mechanisms.

### 4.5. Other Aromatic Compounds

**Figure 5:**
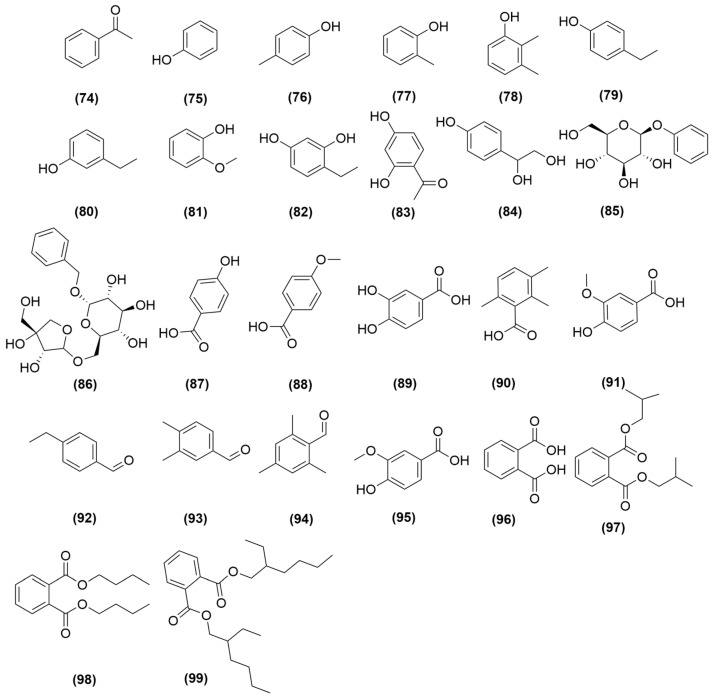
Other aromatic compounds **74**–**99** isolated from Dang Gui.

Furthermore, studies have identified aromatic ketone (**74**), phenolic compounds (**75**–**84**), phenolic glycosides (**85**–**86**), benzoic acid (**87**–**91**), aromatic aldehydes (**92**–**95**), phthalate esters (**96**–**99**) in *A. sinensis*, including acetophenone (**74**), phenol (**75**), p-cresol (**76**), o-cresol (**77**), 2,3-dimethylphenol (**78**), p-ethylphenol (**79**), m-ethylphenol (**80**), guaiacol (**81**), 4-ethylresorcinol (**82**), 2,4-dihydroxyacetophenone (**83**), 4-(1,2-dihydroxyethyl)-phenol (**84**), phenyl-β-D-glucopyranoside (**85**), benzyl-0-β-D-apiofuranosyl-(1-6)-β-D-glucopyranoside (**86**), p-hydroxybenzoic acid (**87**), anisic acid (**88**), protocatechuic acid (**89**), 2,3,6-trimethylbenzoic acid (**90**), vanillic acid (**91**), p-ethylbenzaldehyde (**92**), 3,4-dimethylbenzaldehyde (**93**), 2,4,6-trimeitylbenzaldehyde (**94**), vanillin (**95**), phthalic acid (**96**), bis (2-methylpropyl) phthalate (**97**), dibutp phthalate (**98**), bis (2-ethylhexyl) phthalate (**99**) [37,46,47,48], all of which are illustrated in Figure 5.

### 4.6. Flavonoid Components

**Figure 6:**
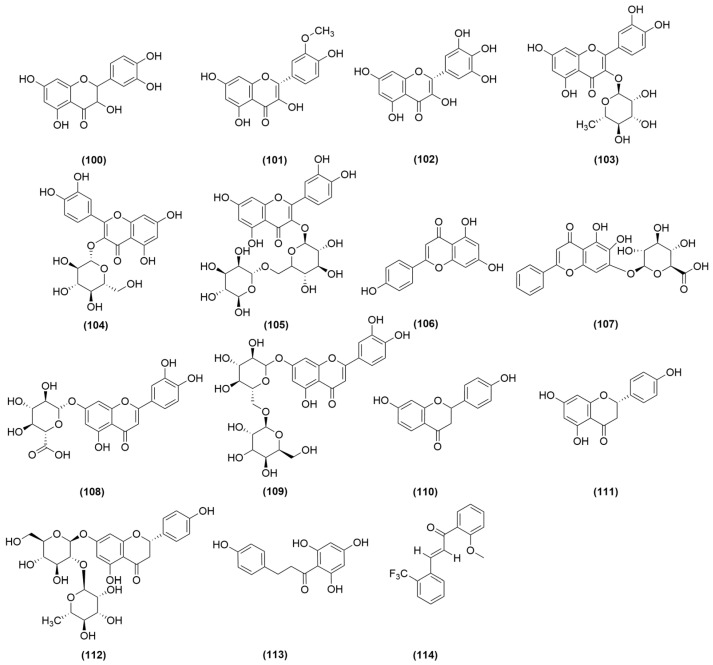
Flavonoid components **100**–**114** isolated from Dang Gui.

Flavonoids (**100**–**114**) represent another class of bioactive constituents in *A. sinensis*, with extraction methods including reflux extraction, ultrasonic extraction and chromatography [49]. Researchers have identified flavonols (**100**–**105**), flavones (**106**–**109**), flavanones (**110**–**112**), chalcones (**113**–**114**) in *A. sinensis*, among which flavonols are the most diverse subclass, including kaempferol (**100**), isorhamnetin (**101**), myricetin (**102**), quercitrin (**103**), isoquercitrin (**104**) and rutin (**105**). Flavones include apigenin (**106**), baicalin (**107**), luteolin-7-O-β-D-glucoside (**108**) and luteolin-7-O-rutinoside (**109**). Flavanones comprise liquiritigenin (**110**), naringenin (**111**) and naringin (**112**). Chalcones include phloretin (**113**), chalcone derivative (**114**) [48,49,50,51], all of which are illustrated in Figure 6.

### 4.7. Other Chemical Constituents

**Figure 7:**
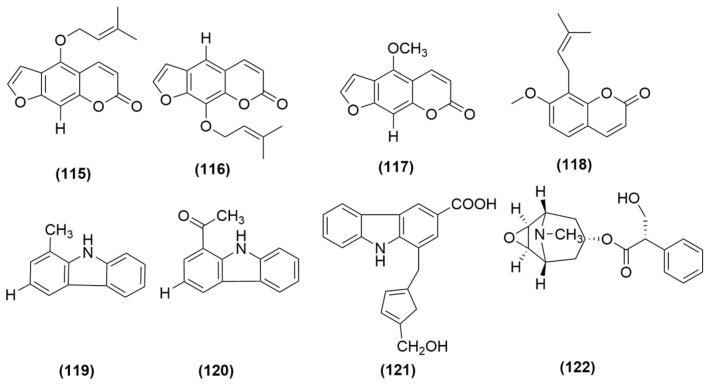
Other chemical components **115**–**122** isolated from Dang Gui.

In addition, studies have demonstrated that *A. sinensis* contains coumarin derivatives (**115**–**118**) and trace amounts of alkaloids (**119**–**122**), among other constituents. The coumarin derivatives include furanocoumarins such as isoimperatorin (**115**), imperatorin (**116**), bergapten (**117**) and osthole (**118**), while the alkaloid components comprise harman (**119**), 1-acetyl-β-carboline (**120**), harmaline (**121**), scopolamine (**122**) [48,52,53] and other analogs, all of which are illustrated in Figure 7.

In summary, *A. sinensis* possesses a complex chemical profile, including polysaccharides, phthalein, phenylpropanoids, terpenoids, and aromatic compounds, flavonoid components, coumarin derivatives and alkaloids, which contribute to its diverse bioactivities. Among these, ASP, (Z)-ligustilide (**3**, Figure 1), and ferulic acid (**38**, Figure 3) are the key immunomodulatory constituents. The identification methods of these chemical constituents are summarized in Table 2. Current research is imbalanced: while polysaccharides are relatively well-studied, most other phthalides, phenylpropanoids, terpenoids, flavonoids and other chemical components remain insufficiently investigated. Polysaccharides also display high structural heterogeneity dependent on extraction and processing, and the structure–activity relationships underlying immunomodulation remain unclear. Future studies should combine advanced analytical techniques with bioactivity-guided fractionation, spectrum–activity relationship analysis, and network pharmacology to clarify the “chemical constituent–immunomodulatory activity” network. This will facilitate the development of efficacy-based quality control standards and mechanism-driven clinical applications of *A. sinensis*.

## 5. Effect of *Angelica sinensis* and Its Components on the Immune System

### 5.1. Restoration of Immune Organ Weights and Their Indices

Research has confirmed that angelica polysaccharides in *A. sinensis* can restore the spleen and thymus indices in animal models. Table 3 presents the effects of ASP on the immune organs. Zhao et al. [31] found that two novel polysaccharides, isolated from *A. sinensis*, designated APS-1a and APS-3a, could elevate the thymus and spleen indices as well as bone marrow cells counts in irradiated mice. These polysaccharides also exhibited protective effects against radiation-induced micronucleus formation in bone marrow cells, indicating their potential application as radioprotective agents. In a separate study, Du et al. [54] studied the protective properties and potential mechanisms of ASP against splenic injury and immune cell damage induced by 5-fluorouracil (5-FU) in mice. The results revealed that ASP treatment mitigated the chemotherapy-induced reduction in spleen weight and organ index, while also improving splenic histopathological structure and functional recovery. Further mechanistic analysis suggested that this protection may be mediated through ASP-induced nuclear translocation of nuclear factor erythroid 2-related factor 2 (Nrf2) and activation of the phosphoinositide 3-kinase/protein kinase B (PI3K/AKT) signaling pathway. Specifically, Nrf2 nuclear translocation induced by ASP alleviates oxidative stress-induced immune cell apoptosis by promoting antioxidant gene expression (e.g., *CAT*, *HO-1*), while PI3K/AKT activation mediated by ASP enhances splenic immune cell proliferation and survival, with such activation only occurring under 5-FU-induced stress rather than normal physiological conditions. Additionally, Yang et al. [55] studied the activity of synthesized angelica APS and its sulfated derivative APS-1 against murine leukemia virus in vivo experiments. Their findings revealed that APS-1 significantly reversed the thymus-to-body weight index reduction induced by viral infection in a dose-dependent manner, not only suppressing viral replication but also enhancing host immune function.

In conclusion, immune organ weight and indices are fundamental for evaluating immune system integrity. Current evidence confirms that ASP exerts radioprotective, antiviral, and immunoregulatory effects by restoring spleen and thymus indices in immune-deficient models; however, its restorative efficacy varies significantly with the injury etiology. Structurally distinct fractions (e.g., APS-1a, APS-3a) show differential potency against radiation- or 5-FU-induced damage, which correlates with ASP structural heterogeneity, the pathological mechanisms of injury (DNA damage/oxidative stress for radiation, cell proliferation inhibition for 5-FU, and complex immune escape for viruses), and administration routes.

Notably, systematic structure–activity relationship studies are lacking, as structural variations (influenced by monosaccharide ratios, linkages, and extraction methods) that affect receptor binding have not been correlated with immune organ protection efficacy. Additionally, non-standardized administration routes (e.g., intraperitoneal injection, gavage) and absent pharmacokinetic data hinder cross-study comparison and clinical translation. Furthermore, the evaluation system is overly narrow, relying solely on organ indices without assessing cellular composition, lymphocyte development, or microenvironmental changes. Research scope is also restricted to central immune organs, leaving protective effects on mucosal-associated lymphoid tissues (e.g., gut, lung) largely unexplored. Therefore, future research should establish a multi-dimensional evaluation system integrating “organ indices—histopathology—cellular function—immune microenvironment,” address the knowledge gap concerning mucosal-associated peripheral immune organs, clarify the structure–activity relationships of structurally distinct ASP and the roles of non-polysaccharide components, and conduct rigorous dose–effect and safety studies to provide the evidence base necessary for clinical translation. At the same time, the effects of non-polysaccharide components (such as ligustilide and ferulic acid) on the central and peripheral immune organs will be systematically evaluated, and research on the synergistic effect of ASP with other active components will be conducted to fill the current research gap and provide a material basis for exploring the “multi-component, multi-target” immune regulation research of *A. sinensis*.

### 5.2. Promoting Proliferation and Activation of Immune Cells

#### 5.2.1. Proliferation and Activation of Macrophages

Macrophages, as tissue-resident or infiltrating immune cells, are pivotal to innate immunity, tissue development, physiological homeostasis, and tissue repair processes [56]. M1 macrophages which migrate to inflammatory sites during host defense, secrete pro-inflammatory cytokines, superoxide anion (O_2_^−^), and nitric oxide (NO).In contrast, M2 macrophages maintain immune homeostasis through the production of anti-inflammatory cytokines and the phagocytic clearance of apoptotic cells [57,58].

Accumulating evidence indicates that polysaccharides derived from *A. sinensis* not only have function as immunological adjuvants to enhance antigen uptake by macrophages and potentiate immune responses, but also can stimulate macrophage proliferation, thereby increasing both the number and functional activity of these cells, as shown in Table 4. Sun et al. [59] studied the effect of ASP on extramedullary erythropoiesis under stress conditions and found that ASP upregulated gene expression of the macrophage chemokine (C-C motif) ligand 2 (*CCl2*) and increased the population of F4/80^+^ macrophages in the mouse spleen. This effect of ASP is achieved through the erythropoietin/signal transducer and activator of transcription 5 (EPO/STAT5) and PI3K/Akt/hypoxia-inducible factor 2α (HIF2α) signaling pathways. Studies have shown that the PI3K/signal transducer and activator of transcription 3 (STAT3) pathway is critical for macrophage phenotypic regulation, in which STAT3 phosphorylation is essential for the function of IL-10, and M2 polarization requires STAT3 mediation to reduce collagen production. Moreover, aging can lead to abnormal activation of this pathway and decreased STAT3 activity, thereby reducing cardiac M2 macrophage infiltration and impairing cardioprotection. N-butylphthalide (**5**, Figure 1), a constituent of the volatile oil of *A. sinensis*, can target the PI3K/STAT3 pathway, promote M2 macrophage polarization, and alleviate myocardial fibrosis. Meanwhile, this phthalide can also regulate multiple key targets along the PI3K/STAT3 cascade, including promoting the phosphorylation of STAT3, and activating its downstream anti-inflammatory and anti-fibrotic signaling, thus further enhancing the phenotypic transformation of macrophages and protecting cardiac function [60].

In the mouse tumor model, Shen et al. [61] found that ASP can upregulate monocytes/macrophages, T cells, cytotoxic T lymphocyte (CD8^+^T cells), gamma delta T cells, and granulocytes in both peripheral blood and spleen of mice, with effects showing a dose-dependent pattern. Meanwhile, in vivo studies confirm that STAT3 is critical for myeloid-derived suppressor cell (MDSC) proliferation. STAT1 modulates arginase and inducible Nitric Oxide Synthase (iNOS) activity in M-MDSCs and inhibits IFN-γ secretion by T cells. APS dose-dependently elevates the phosphorylation levels of STAT1 and STAT3 in MDSCs, which may enhance MDSC function via regulating these two pathways. Furthermore, toll-like receptors (TLRs) are a family of transmembrane proteins in mammals that play a key role in innate immune recognition. Yang et al. found that ASP can enhance the mRNA expression of toll-like receptor 4 (*TLR4*), thereby activating the TLR4 signaling pathway to promote the release of NO, TNF-α and ROS by macrophages and increase the activities of iNOS and LSZ in macrophages [62]. Chen et al. [63] compared the immunomodulatory effects of ASP, *A. sinensis* oligosaccharides, *A. sinensis* sucrose, and total amino acids of *A. sinensis* on mouse peritoneal macrophages. The results demonstrated that ASP significantly enhanced macrophage growth and proliferative activity and exhibited synergistic immunomodulatory potential when combined with total amino acids, and this effect of ASP might be associated with the upregulation of *TLR4* mRNA. The effect of the active components of *A. sinensis* in promoting the proliferation and activation of lymphocytes is shown in Table 4.

**Table 4:** Promoting effect of active components from *A. sinensis* on proliferation and activation of immune cells.

No.	Chemical Component	Model	Pathways	Effects	Ref.
1	ASP	In vivo: stress-induced anemia mouse model	EPO/STAT5 PI3K/Akt/HIF2α	↑ cellular proliferation, CCl2, F4/80^+^ macrophages, splenic macrophages, ↓ IL-1β	[59]
2	n-butylphthalide (**5**, Figure 1)	In vivo: myocardial infarction rat model	PI3K/STAT3	↑ M2 macrophages, M2c subtype macrophages, IL-10 ↓ M1 macrophages	[60]
3	ferulic acid (**38**, Figure 3)	In vivo: hematopoietic cell depletion mouse model	/	↑ white blood cells ↑ CFU-GEMM	[64]
4	ASP	In vivo: mouse tumor model In vitro: MDSC model	STAT1 STAT3	↑ CD8^+^ T cells, T cells, NK cells, γδT cells, granulocytes, MDSC	[61]
5	AAP	In vivo: female BALB/c mouse model In vitro: mouse peritoneal macrophage model	TLR4	↑ macrophages enzyme activity, NO, TNF-α, TLR4 mRNA	[62]
6	AP I AP II	In vivo: Balb/c strain of mice model In vitro: mouse splenic lymphocyte model	/	↑ lymphocyte, IFN-γ	[65]
7	ligustilide	In vivo: acute thymus atrophy mouse model In vitro: mouse iTECs model	Tβ15-G-actin	↑ thymic epithelial cells, CD4SP T cells ↓ thymic damage	[66]
8	ASP	In vitro: mouse peritoneal macrophage model	*TLR4* mRNA	↑ macrophages ↑ iNOS, LZM, ICAM-1, TLR4 mRNA	[63]
9	ASP-PLGA	In vitro: mouse splenic lymphocyte model	/	↑ lymphocytes, CD4^+^/CD8^+^ T cells	[67]
10	ASP	In vitro: mouse splenic lymphocyte model	/	↑ macrophages, lymphocyte	[68]
11	sCAP	In vitro: chicken peripheral blood lymphocyte	/	↑ lymphocyte	[69]
12	AP-3	In vitro: mouse splenic cell	/	↑ macrophages, mixed lymphocyte, IFN-γ, IL-2	[70]
13	CAPS_30_, CAPS_50_, CAPS_70_, CAPS_80_	In vitro: peripheral blood lymphocyte	/	↑ lymphocytes, IFN-γ, IL-2, IL-6, TNF–α ↑ CD3^+^, CD56^+^ cells	[32]

Abbreviations: EPO/STAT5, erythropoietin/signal transducer and activator of transcription 5; PI3K/Akt/HIF2α, phosphoinositide 3-kinase/protein kinase B/hypoxia-inducible factor 2α; PI3K/STAT3, phosphoinositide 3-kinase/signal transducer and activator of transcription 3; TLR4, toll-like receptor 4; MDSC, myeloid-derived suppressor cell; STAT1, signal transducer and activator of transcription 1; CFU-GEMM, colony-forming unit-granulocyte-erythrocyte-monocyte-megakaryocyte; iTECs, immature thymic epithelial cells; Tβ15-G-actin, thymosin β15-G-actin; CD4SP T cells, CD4+ single-positive T lymphocytes; iNOS, inducible nitric oxide synthase; LZM, lysozyme; ICAM-1, intercellular adhesion molecule-1; PLGA, polylactic-co-glycolic acid; sCAP, selenium-modified Angelica polysaccharide; AP-3, *Angelica* polysaccharide-3; CAPS30/50/70/80, graded Angelica polysaccharides (30/50/70/80); CD3^+^/56^+^, cluster of differentiation 3^+^/56^+^ cells; NK cells, natural killer cells. ↑ means increase; ↓ means decrease.

#### 5.2.2. Proliferation and Activation of Lymphocytes

Shan et al. [65] found that various ASP fractions could enhance the proliferation and biological activity of mouse spleen lymphocytes both in vitro and in vivo, and simultaneously stimulate the production of interferon-γ (IFN-γ) and other cytokines, thereby regulating the cellular immune response. And studies have shown that encapsulating ASP into polylactic acid glycolic acid copolymer (PLGA) to form ASP-PLGA can significantly promote lymphocyte proliferation and enhance immune function [67]. Moreover, the extraction methods applied to ASP can influence its capacity to stimulate T cell proliferation to varying degrees [68]. Qin et al. [69] found that selenium-modified ASP, obtained via water decoction and ethanol precipitation, further augments the immunomodulatory activity of Chinese angelica polysaccharide by promoting the proliferation of peripheral lymphocytes in chickens. In a comparative study, Wang et al. [32] extracted four graded polysaccharides from *A. sinensis*, named CAPS_30_, CAPS_50_, CAPS_70_, and CAPS_80_, which were evaluated for immunostimulatory activity in vitro. All four were found to induce lymphocyte proliferation and upregulate secretion of IFN-γ, tumor necrosis factor-α (TNF–α), interleukin (IL)-2, IL-6. Among them, CAPS_50_ and CAPS_70_ also increased the proportion of cluster of differentiation 3 (CD3^+^) T lymphocytes.

In addition, Deng et al. [64] found that ferulic acid (**38**, Figure 3) in *A. sinensis* can promote the recovery of white blood cell count in a mouse model of blood cell deficiency. Xu et al. [66] found that ligustilide in the volatile oil of *A. sinensis* can reverse thymus damage, promote thymic epithelial cell (TEC) proliferation and reticular differentiation, increase the proportion of CD4^+^ single positive (CD4SP) T cells, and alleviate thymic immune aging induced by doxorubicin. This effect of ligustilide is achieved by inhibiting the binding of thymosin β15 (Tβ15) to G-actin. Yang et al. [70] found that ASP can activate macrophages and T cells, and can also exert immunomodulatory effects through cytokines IL-2 and IFN-γ.

In summary, immune cell proliferation and activation are core steps in initiating immune responses, and active components of *A. sinensis* promote this process via TLR4-related signaling. Macrophages are key targets: ASP may activate macrophages through TLR4, and via the EPO/STAT5 pathway in stress-induced anemia to facilitate erythroid differentiation. Notably, distinct components exert divergent effects—ASP primarily promotes M1 polarization, while volatile oil-derived butylphthalide induces M2 polarization via PI3K/STAT3 to alleviate myocardial fibrosis in rats. Mechanistically, macrophage surface TLR4 and Dectin-1 are critical for polysaccharide recognition, with ASP structural characteristics potentially determining receptor selectivity; however, direct validation of receptor-mediated macrophage proliferation and comparative studies on receptor selectivity of different ASP structures are lacking.

Lymphocytes, as core executors of adaptive immunity, are significantly regulated by *A. sinensis* components. Various ASP fractions and graded CAPS_30–80_ (with CAPS_70_ most potent) promote splenic lymphocyte proliferation and induce IFN-γ/IL-2 secretion in vitro and in vivo. Ligustilide maintains the thymic microenvironment via Tβ15 to support T cell development, while preparation/modification methods strongly influence ASP activity—microwave-dried polysaccharides enhance lymphocyte proliferation (unlike far-infrared-dried counterparts), and selenylated/sulfated ASP further boost lymphocyte function, inhibit viral replication, and improve host immunity.

Nevertheless, current research faces critical bottlenecks: over 90% of studies focus on macrophages and lymphocytes, leaving dendritic cells (DC), natural killer (NK) cells, and mast cells largely unexplored. Core mechanisms suffer from logical gaps—most studies only detect activation phenotypes without validating immune cell activation as a necessary condition for efficacy via cell depletion or conditional knockout. In vitro monoculture experiments outnumber vivo studies, with the validity of in vitro conclusions in complex in vivo microenvironments unconfirmed. Additionally, component-specific regulatory preferences for immune subsets and multi-component synergies remain unelucidated. Future research should fill gaps in non-dominant immune cells, validate the core driving role of immune activation via lineage tracing/knockout, establish physiologically relevant co-culture/organoid models, and clarify component specificity and multi-component synergies.

### 5.3. Inhibiting Excessive Activation of Immune Cells

However, excessive activation of immune cells, including macrophages, mast cells, and T helper 2 (Th2) cells, can trigger inflammation responses, oxidative stress, and other pathological reactions. The effective components of *A. sinensis* effectively inhibited the excessive response of immune responses, as displayed in Table 5. Cheng et al. [71] revealed that ferulic acid (**38**, Figure 3) can suppress the dominant expression of Th2 related factors in a membranous nephropathy (MN) rat model, which may be related to the regulation of oxidative stress, angiogenesis and anti-angiogenic factors, as well as the suppression of Th2-mediated immune response. Proto-oncogene tyrosine–protein kinase (Fyn) is a crucial signaling molecule for activation of mast cells stimulated by various antigens. Mao et al. [72] demonstrated that ASP can downregulate the activation of mast cells by suppressing pro-inflammatory cytokines and allergens release, as well as downregulating GRB2-associated binding protein 2 (Gab2)/PI3K/Akt and Fyn/spleen tyrosine kinase (Syk) signaling pathways. Fu et al. [73] found that n-butylphenolphthalein, a phthalein compound also derived from *A. sinensis*, can significantly reduce the secretion of IL-6 and TNF-α in lipopolysaccharide (LPS)-stimulated DC2.4 in mice, and this inhibitory effect is achieved by suppressing the nuclear factor kappa-B (NF-κB) signaling pathway. In the experiment of excessive proliferation of spleen cells induced by concanavalin A (Con A), it was found that ASP could significantly inhibit the proliferation of spleen cells and CD4^+^T cells, while promoting the proliferation of CD19-positive B (CD19^+^B) cells, thereby achieving a bidirectional regulatory effect on immune cells [74].

Building on these mechanistic insights, it becomes evident that different active components of *A. sinensis* target distinct nodes of the immune network: ferulic acid modulates adaptive immunity by suppressing Th2 responses; n-butylphthalide acts on dendritic cells to curb NF-κB-driven inflammation; ASP exerts dual effects on innate and adaptive immunity by inhibiting mast cell activation while fine-tuning T/B cell balance. This multi-component, multi-target regulatory network enables *A. sinensis* to achieve precise immunomodulation without tipping into global immunosuppression—a hallmark advantage of herbal therapeutics. This precise regulation stems from the synergy of multiple components in *A. sinensis*—different components target distinct nodes of the immune response to jointly maintain immune homeostasis. However, current research has obvious limitations: most studies remain at the phenotypic description stage without validating direct molecular targets; the boundary between inhibiting excessive activation and comprehensive immunosuppression is unclear; and the synergistic mechanism among different components has not been systematically evaluated. Future research should focus on three aspects: verifying key targets using gene knockout models; elucidating the cell subset specificity of component effects via single-cell technology; and exploring the molecular network of multi-component synergistic regulation of immune homeostasis, so as to provide a theoretical basis for the selective intervention of inflammatory diseases.

### 5.4. Modulation of Immune Active Substance Release

Many studies found that the primary bioactive components from *A. sinensis* can modulate the release of the immune active substances, as shown in Table 6. NF-κB is a key transcription factor mediating the production of pro-inflammatory cytokines and chemokines linked to inflammation and autoimmune diseases, and blocking its activation may ameliorate Con A-induced liver injury. The IL-6/STAT3 signaling pathway not only mediates inflammatory responses but also leads to a significant increase in STAT3 phosphorylation levels [75].In a Con A-induced mouse model of liver injury, ASP can significantly reduce pro-inflammatory cytokines IFN-γ, TNF-α, IL-2, and IL-6, and this anti-inflammatory effect of ASP was achieved through the inhibition of the IL-6/STAT3 and NF-κB signaling pathways [74]. Li et al. [76] found that ASP can alleviates inflammation in complete Freund’s adjuvant (CFA) induced arthritic rats by inhibiting TNF-α and IL-6 levels and suppressing the IL-6/janus kinase 2 (JAK2)/STAT3 signaling pathway. In a murine model of sepsis, ASP was shown to reduce myeloperoxidase (MPO) activity—a marker of neutrophil infiltration—and inhibit TNF-α, IL-6, IL-18, and IL-1β expression, thereby protecting against sepsis-induced acute lung injury through inhibition of the NOD-like receptor family pyrin domain containing 3 (NLRP3) and NF-κB pathways [77]. In the acute inflammation rat model, it was found that the volatile oil of *A. sinensis* could reduce the levels of white blood cell (WBC), neutrophil percentage (NE%), TNF-α, NO, inducible nitric oxide synthase (iNOS), cyclooxygenase-2 (COX-2), IL-1β, and IL-6, exerting liver-protective and anti-inflammatory properties [78]. Zhong et al. [79] compared processing methods and found that stir-fried and alcohol-processed the volatile oil of *A. sinensis* exhibited the strongest inhibition of PGE_2_, histamine, 5-hydroxytryptamine, and TNF-α release. Furthermore, urine metabolomics studies have revealed that disrupted metabolism of glycine, arachidonic acid, L-glutamate, pyruvate, and succinate may play a critical role in the susceptibility and progression of LPS-induced inflammation. Notably, the volatile oil of *A. sinensis* can regulate these metabolic disturbances to exert anti-inflammatory effects [80]. Li et al. [81] not only found multiple types of ligustilide in the volatile oil of *A. sinensis*, but also found in a mouse model of ear edema that *A. sinensis* volatile oil can significantly reduce the levels of inflammatory cytokines TNF-α, COX-2, and IL-6.

In the model of hepatic stellate cell, Wang et al. [82] revealed that ASP can significantly enhance the secretion of IL-22 in splenic cells and hepatic tissues. And this effect of ASP can effectively alleviate the liver fibrosis condition of mice with chronic liver fibrosis, thereby suppressing activation of hepatic stellate cell (HSC) via the IL-22/signal transducer and STAT3 pathway. Chao et al. [83] demonstrated that the ethyl acetate extract of *A. sinensis*, rich in ferulic acid (**38**, Figure 3), ligustilide and other components, can significantly inhibit the NF-κB luciferase activity in LPS/IFN-γ and IL-6-stimulated RAW 264.7 cells, and reduce the secretion of TNF-α. In addition, it can suppress the release of NO and macrophage inflammatory protein-2 (MIP-2)—a chemokine whose expression is dependent on NF-κB activation, thereby alleviating acute inflammatory injury. Choi et al. [84] found that glabralactone, a coumarin derivative from *A. sinensis* with a structure analogous to compounds **38**–**50**, acts as an effective inhibitor of NO release in macrophages, and this inhibitory effect is mediated through the suppression of the NF-κB and TIR-domain-containing adaptor protein inducing IFN-β- (TRIF-) dependent interferon regulatory factor 3 (IRF-3) pathways.

Su et al. [85] further identified that ligustilide in *A. sinensis* can prevent LPS induced iNOS, NO, PGE_2_ and TNF-α expression in RAW 264.7 macrophages by scavenging reactive oxygen species (ROS) generation and downregulating the mitogen-activated protein kinase (MAPK), activator protein-1 (AP-1) and NF-κB signaling pathways. Meanwhile, Chen et al. [86] found that ferulic acid (**38**, Figure 3) from *A. sinensis* reduced hydrogen peroxide induced TNF-α, IL-1β, MMP-1, and matrix metalloproteinase 13 (MMP-13) expression, and upregulated SRY-box transcription factor 9 (*SOX9*) gene to protect osteoblasts.

In inflammatory or immune hyperactivity states, immune homeostasis is imbalanced—characterized by upregulated pro-inflammatory mediators (e.g., IL-2, IL-6, TNF-α, IFN-γ) and downregulated regulatory cytokines (e.g., IL-10, IL-22). Active components of *A. sinensis* (polysaccharides, ferulic acid, volatile oils) restore balance by inhibiting excessive pro-inflammatory mediator expression, via regulating key pathways (STAT3, NF-κB, Caspase-8, NLRP3) and metabolisms (histidine, tryptophan, fatty acid).

Notably, components act on distinct targets: ASP inhibits mast cell activation via Gab2/PI3K/Akt and Fyn/Syk, ligustilide maintains the thymic microenvironment by modulating thymosin β15; butylphthalide induces M2 macrophage polarization through PI3K/STAT3 to exert anti-inflammatory effects. These components regulate cytokine networks—IL-2 modulates T cell proliferation, TNF-α enhances phagocytosis and T/B cell differentiation, and IL-6 supports T cell development and antibody production—enabling precise modulation of immune cell functions.

**Figure 8:**
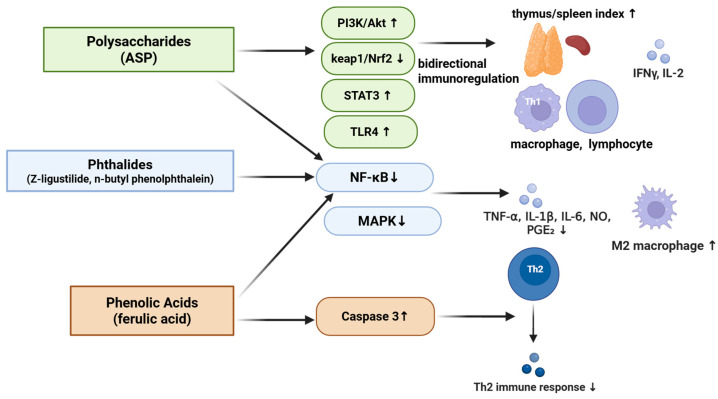
The immunomodulatory effect of the active components of *A. sinensis.* Created in BioRender. Li, T. (2026) https://BioRender.com/6z0osak (accessed on 24 March 2026). (↑ means increase; ↓ means decrease).

However, critical mechanistic gaps persist. Most studies only detected changes in immune-active substances, but failed to determine their cellular origin, thus failing to clarify the cascade reaction between the active components in *A. sinensis*, immune cells, immune-active substances and the therapeutic effect. The driving role of immunomodulation requires further causal validation. Research remains phenotypic, focusing on individual factor changes rather than cytokine network hierarchies, and is homogenized—concentrating on classical pro-inflammatory cytokines (IL-1β, IL-6, TNF-α) while neglecting emerging regulators (IL-10, IL-22). Future research should use cell sorting and conditional knockout to confirm cellular sources of immune-active substances and immunomodulation’s core role, employ multi-omics to dissect cytokine networks, and fill gaps in emerging regulatory cytokines via loss-of-function experiments to complete the mechanistic landscape. The immunomodulatory effects achieved through multiple pathways and multiple targets by compounds such as ASP are as shown in Figure 8.

## 6. Pharmacological Effects Through Immune Mediation

### 6.1. Anti-Tumor

#### 6.1.1. Regulating Expression of Cytokines

Studies have confirmed that multiple cytokines, including transforming growth factor-β (TGF-β), interleukin-6 (IL-6), and vascular endothelial growth factor (VEGF), play critical roles in tumor cell proliferation and metastasis. The active components of ASP can modulate cytokine expression, thereby exerting anti-tumor effects.

In colorectal cancer research, supercritical fluid extracts of *A. sinensis*, including n-butylphthalide (**5**, Figure 1), ferulic acid (**38**, Figure 3), caffeic acid (**40**, Figure 3) and chlorogenic acid (**42**, Figure 3), can suppress the expression of inflammatory mediators such as inducible nitric oxide synthase (iNOS) and COX-2 in tumor tissues, suggesting that *A. sinensis* extract may interfere with the initiation and progression of colorectal cancer [87]. On the other hand, Gao et al. [88] found that *A. sinensis* could inhibit the enzymatic activities of MMP-2 and MMP-9, reduce the levels of cytokines such as TGF-β1, and suppress the invasive and migratory capabilities of A549 human lung adenocarcinoma cells, indicating its potential in inhibiting tumor metastasis. Furthermore, Ren et al. [89] confirmed that ASP not only inhibits iron accumulation in the liver, spleen and transplanted tumors of tumor-bearing mice but also significantly downregulates the hepatic expression of IL-6, JAK2 and p-STAT3, p-SMAD1/5/8. Furthermore, this study demonstrated that ASP can suppress hepcidin expression and iron deposition in tumor-bearing mice by inhibiting the JAK/STAT and bone morphogenetic proteins (BMP)/Sma- and Mad-related protein (SMAD) signaling pathways, thereby inhibiting tumor proliferation. In vitro experiments in mouse peritoneal macrophages, Yang et al. [90] investigated the effect of ASP on the production of effector molecules in peritoneal macrophages and found that ASP can promote the release of NO, TNF-α and reactive oxygen species (ROS), and enhance the activities of inducible iNOS and lysozyme (LSZ), thereby exerting indirect anti-tumor effects.

#### 6.1.2. Regulating Immune Cells and Tumor Immune Microenvironment

Immune cells including tumor-associated macrophages (TAMs) and regulatory B cells (Bregs) are associated with the prognosis of bladder cancer, gastric cancer and other malignancies [91]. Within the tumor microenvironment, the suppression of Th1-type immune responses and the concomitant enhancement of Th2-type responses lead to impaired anti-tumor immunity [92]. The inhibitory effects of *A. sinensis* on tumor cells via the immune system is shown as Table 7.

**Figure 9:**
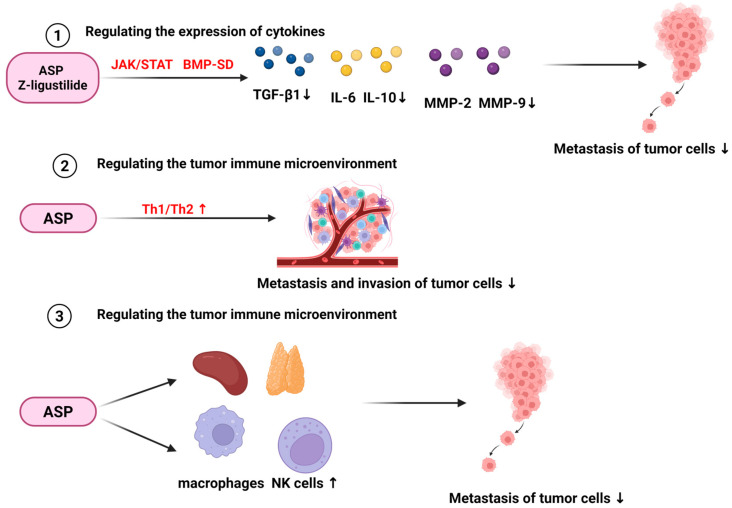
Regulatory effect of active components chemical components on tumor cells through the immune pathway. Created in BioRender. Li, T. (2026) https://BioRender.com/a92nuq2 (accessed on 24 March 2026). (↑ means increase; ↓ means decrease).

Notably, a novel alkaline-soluble polysaccharide (AASP) has been identified in tumor-bearing mice. This polysaccharide not only protects the spleen and thymus from damage but also exerts stimulatory effects on the proliferation of macrophages, splenocytes, NK cells and other immune cells [93]. Shang et al. [94] demonstrated that total polysaccharides extracted from *A. sinensis* can significantly alleviate thymic atrophy in sarcoma-bearing mouse models. Hao et al. [95] found that A-SFE, which contains (Z)-ligustilide (**3**, Figure 1), could directly inhibit the proliferation, migration and invasion of colorectal cancer cells in vitro, and also suppress the M2 polarization of tumor-associated macrophages to regulate the tumor microenvironment. The combined use of A-SFE and oxaliplatin exhibited synergistic or additive anti-proliferative effects on colorectal cancer cells in vitro, and their combination significantly reduced tumor volume and weight in a mouse CT26 syngeneic xenograft model, while downregulating the expression of Ki67, MMP9 and CD206 in tumor tissues. Furthermore, the research has confirmed that the ASP released from AP-PP-DOX can upregulate the expression of IL-2 and downregulate the expression of IL-10, indicating its potential to restore the Th1/Th2 balance and its ability to regulate the tumor microenvironment [96]. A schematic illustration of how the bioactive components of *A. sinensis* inhibit tumor cell proliferation and metastasis by modulating immune cells, cytokines, and the tumor immune microenvironment is presented in Figure 9.

In the clinical treatment of cancer, chemotherapeutic agents such as cisplatin, carboplatin and cyclophosphamide can induce gastric mucosal damage. Studies have shown that subcutaneous administration of ASP can promote the recovery from leukopenia and increase the density of blood vessels and proliferating cells in gastric and duodenal tissues, thereby exerting a protective effect on the gastrointestinal tract [97].

In summary, ASP can exert anti-tumor effects through a variety of interrelated mechanisms: they regulate the JAK/STAT signaling pathway, downregulate the expression of IL-6 and TNF-α, alleviate hepcidin-mediated iron homeostasis imbalance, restore hepatic iron metabolic balance, and thereby inhibit tumor progression driven by iron overload. Meanwhile, ASP and other active components of *A. sinensis* exert immunomodulatory effects by targeting the inflammatory cytokine cascade and maintaining the metabolic homeostasis of the tumor microenvironment. However, as key markers of immune activity, the exact roles of iNOS and NO and the regulatory mechanisms of *A. sinensis* components on them remain unclear.

Despite the substantial accumulation of experimental data on the immunomodulatory effects of *A. sinensis*, the field remains largely at the stage of “phenomenon accumulation” rather than “mechanistic elucidation” and “systematic integration”. Core limitations are evident in four aspects. First, mechanistic depth is insufficient. Most studies present linear descriptions of components exerting effects through pathways but lack identification and validation of direct molecular targets. For instance, while ASP inhibits mast cell activation and regulates JAK/STAT signaling, the systematic analysis of JAK/STAT upstream activators (e.g., gp130, SOCS) and downstream transcriptional targets remain unexplored. Second, polysaccharides have been relatively intensively studied, but dozens of terpenoids in volatile oils, aside from ligustilide and butylphthalide, remain almost unexplored. The receptor selectivity of structurally diverse ASP and their structure–activity relationships with immune effects have not been systematically established. Third, synergistic or antagonistic interactions among multiple components have not been systematically evaluated, hindering explanation of the therapeutic advantages of *A. sinensis* compound preparations. Moreover, the emerging dimension of immunometabolism—the crosstalk between immune cells and metabolic pathways in the tumor microenvironment—has not been incorporated into research scope. Fourth, the development of nano-delivery systems loaded with *A. sinensis* active components remains nascent, lacking pharmacokinetic and systemic toxicological data, which severely limits clinical translation.

Future research must transcend reductionist single-component, single-pathway thinking by integrating systems pharmacology, chemical biology, single-cell technologies, and gene editing tools. Priority areas include identifying direct molecular targets of key components; elucidating receptor selectivity and structure–activity relationships of structurally diverse ASP; revealing immunometabolic regulatory networks through integrative omics; exploring molecular mechanisms of multi-component synergy; and establishing clinically aligned pharmacological evaluation systems and nano-delivery platforms. Such efforts will advance the field from fragmented phenotypic description toward systematic mechanistic elucidation, laying a scientific foundation for precise clinical application and international development of *A. sinensis*.

### 6.2. Regulate Hematopoiesis

#### 6.2.1. Regulating the Expression of Immune Cells and Their Ability to Form Colonies

Studies have revealed that the pathogenesis of various types of anemia is closely associated with macrophages, natural killer (NK) cells and T lymphocytes [98,99,100]. In the model of stress-induced anemia, Wang et al. [101] demonstrated that ASP not only significantly accelerates the recovery of red blood cells, hemoglobin and hematocrit in peripheral blood, but also increases the number of F4/80^+^VCAM-1^+^ central macrophages in splenic erythroid islands. Their research results indicate that ASP may promote the generation and differentiation of red blood cells by enhancing the sensitivity of peripheral hematopoietic organs to erythropoietin (EPO), and by enhancing the functions mediated by macrophages, including adhesion, iron homeostasis, cell migration and erythrocyte nuclear exocytosis.

And in acute hemorrhagic mice model, researchers found that low-dose water-soluble polysaccharides from *A. sinensis* can regulate the expression of IL-6 and IL-3, and enhance the colony-forming ability of granulocyte–macrophage (GM) progenitors [102]. Chen et al. [103] conducted clinical studies and demonstrated that *A. sinensis* administration was significantly associated with a reduced incidence of transfusion dependence in patients with aplastic anemia (AA). This therapeutic benefit may be attributed to its ability to suppress IL-17 expression and ameliorate anemia symptoms. Subsequent in vitro and in vivo experiments further revealed that ASP effectively attenuates p38/MAPK signaling pathway-mediated mitochondrial apoptosis, decreases the proportion of activated Th17 cells and inhibits IL-17 expression. In anemic patients and cancer patients undergoing radiotherapy and chemotherapy, acidic polysaccharides from *A. sinensis* exert a protective effect on bone marrow hematopoietic function and CD34^+^ progenitor cells by stimulating the secretion of granulocyte–macrophage colony-stimulating factor (GM-CSF) and IL-3 in peripheral blood [104].

#### 6.2.2. Regulating the Expression of Cytokines

Tian et al. [105] found that ASP can regulate the levels of IL-3 and granulocyte colony-stimulating factor (G-CSF) while reducing TNF-α levels in a rat model of blood deficiency, thereby promoting hematopoiesis via cytokine regulation. This provides further evidence that the aqueous extract of *A. sinensis* enhances hematopoietic function through immune modulation. Furthermore, Chang et al. [106] found in a rat model of severe exercise-induced anemia that the root extract of *A. sinensis,* containing ferulic acid (**38**, Figure 3), (Z)-ligustilide (**3**, Figure 1), etc., not only significantly increases the red blood cell count and hemoglobin concentration in anemic rats, but also reduces hepatic IL-6 expression, demonstrating that the root of *A. sinensis* exerts a protective effect against exercise-induced anemia.

In addition, the majority of iron required for erythropoiesis is derived from the phagocytic recycling of senescent red blood cells by macrophages [107]. Inflammatory cytokines directly disrupt iron homeostasis by stimulating the synthesis of divalent metal ion transporter 1 (DMT1) in macrophages, upregulating ferritin expression while impairing its biological function, enhancing transferrin receptor (TfR)-mediated iron uptake, and promoting erythrophagocytosis [108]. Studies demonstrated that ASP can mitigate the inflammatory perturbation of iron metabolism. Specifically, ASP inhibits hepatic hepcidin expression by suppressing the phosphorylation of STAT3 and SMAD 1/5/8 and concurrently upregulates ferroportin expression in the spleen and liver to facilitate iron efflux from these iron storage organs into the plasma. Moreover, ASP can not only downregulate the expression of iron storage and transport proteins, including ferritin, DMT1 and TfR1, but also reduce white blood cell counts and the mRNA expression of pro-inflammatory cytokines *IL-1β*, *TNF-α*, and IL-6. These actions collectively enhance EPO production in the kidney and liver, improve systemic iron bioavailability, and restore erythropoietin receptor (EPOR) signaling, thereby ameliorating anemia in a rat model of chronic kidney disease (CKD) [109]. Wang et al. further found that acidic polysaccharides from *A. sinensis* can inhibit NF-κB p65 activation via the IκB kinase (IKK)-IκBα pathway in rats with anemia of chronic disease (ACD), thereby reducing the secretion of IL-6 and TNF-α and effectively alleviating anemia [110]. The active components of *A. sinensis* have an effect on improving hematopoietic function through the immune system as shown in Table 8.

**Figure 10:**
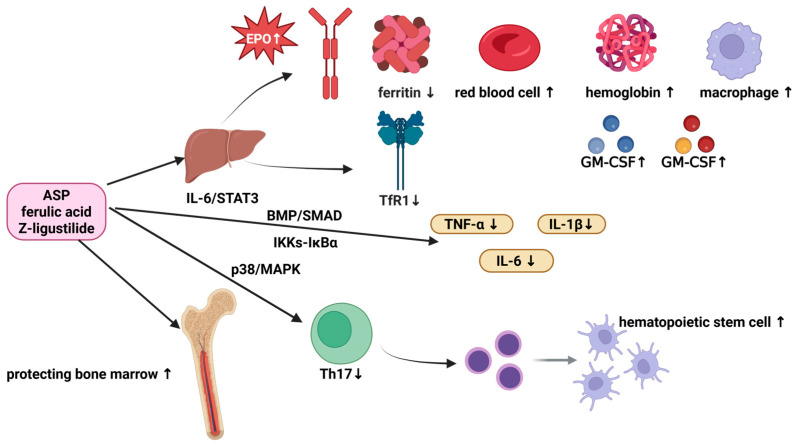
Regulatory effect of active components from *A. sinensis* on hemopoietic system through the immune pathway. Created in BioRender. Li, T. (2026) https://BioRender.com/r8xwjsb (accessed on 24 March 2026). (↑ means increase; ↓ means decrease).

Recent studies have explored the use of ASP as a biopolymer for modulating the size and stability of iron oxide nanoparticles (IONPs), resulting in the development of a nanoplatform designated IONPs@ASP. In anemic model rats, administration of IONPs@ASP restored hemoglobin levels to the normal range. Mechanistic analysis revealed that ASP upregulated the expression of anti-inflammatory factors, suggesting a synergistic mechanism integrating iron supplementation with immunomodulation in this animal model. These findings not only represent a significant advancement in anemia therapy but also lay a solid foundation for the development of natural polysaccharide-based multifunctional nanomedicines [111]. The schematic diagram of how the active components of *A. sinensis* promote red blood cell production and regulate iron metabolism by regulating immune cells, immune active substances, etc., is shown in Figure 10.

In summary, *A. sinensis* active components exert hematopoietic effects through an immune regulatory network, with ASP as the primary mediator acting on macrophages, hematopoietic progenitors, and cytokine networks. Ferulic acid (**38**, Figure 3) and (Z)-ligustilide (**3**, Figure 1) promote erythrocyte recovery (mechanisms unclear), while other volatile oil components remain understudied. At the cellular level, ASP enhances splenic F4/80^+^VCAM-1^+^ central macrophage proliferation, strengthens erythroblastic island function, and increases granulocyte–macrophage progenitor colony-forming capacity and CD34^+^ cell numbers. At the molecular level, it regulates EPO/STAT5, p38/MAPK, IL-6/STAT3 pathways, modulates hematopoietic factors (IL-3, GM-CSF) and pro-inflammatory cytokines (TNF-α, IL-1β), and improves iron utilization via hepcidin, DMT1, and TfR1—collectively forming an “immune–hematopoiesis” coupled mechanism.

Despite these advances, current research exhibits several notable deficiencies. The core regulatory relationships remain ambiguous, with it still unclear whether ASP acts directly on hematopoietic stem/progenitor cells or indirectly through modulation of the hematopoietic microenvironment and immune cells—the hierarchical logic and upstream–downstream relationships governing these effects have yet to be elucidated. The research scope is narrowly focused, predominantly concentrating on erythroid hematopoiesis while largely neglecting regulatory effects on myeloid and lymphoid lineages, thereby failing to comprehensively explain the clinical hematopoietic benefits observed. Pathological relevance is insufficient, with most studies employing acute anemia models while investigations into chronic disease anemia and cancer-related anemia—the most common clinical forms—remain limited, particularly regarding stage-specific heterogeneity. Translational research lags behind, with optimal dosing regimens and dose–response relationships for different anemia subtypes remaining undefined, and standardized preclinical safety data lacking. Future research should employ lineage tracing and cell-specific knockout technologies to clarify the core regulatory logic of “immune–hematopoietic” coupling, address the knowledge gap concerning myeloid and lymphoid hematopoiesis, establish clinically relevant chronic anemia models to define stage-specific intervention effects, and conduct rigorous dose–response and long-term safety studies to provide the evidence base necessary for precise clinical application.

### 6.3. Anti-Osteoarthritis by Regulating Cytokines

Rheumatoid arthritis (RA) is a chronic autoimmune disorder characterized by joint inflammation, in which the suppression of key inflammatory mediators represents a cornerstone of therapeutic intervention. Interleukin-6 receptor (IL-6R) signaling is a key pathogenic mechanism underlying RA [112]. And extracts of *A. sinensis* have been shown to suppress receptor activator for nuclear factor-κB ligand (RANKL)-induced osteoclast differentiation in bone marrow-derived macrophages (BMMs) in a dose-dependent manner. This effect was accompanied by marked inhibition of phosphorylation in MAPK signaling components such as p38, extracellular signal-regulated kinase (ERK), c-Jun N-terminal kinase (JNK) and the NF-κB pathway (p65 phosphorylation and IκB degradation), suggesting its efficacy in preventing inflammatory bone resorption [113]. In rat models of arthritis, ASP can inhibit the expression of IL-6, IL-1β, iNOS, MMP-1 and MMP-3, as well as reduce the proliferation, migration and invasion of TNF-α-induced fibroblast-like synoviocyte (FLS) cells. ASP also promotes FLS apoptosis, leading to reduced paw swelling, lower arthritis scores and suppressed synovial hyperplasia. Further mechanistic studies have revealed that ASP can modulate the gut microbiota and the JAK2/STAT3 signaling pathway to ameliorate RA symptoms [114]. Another study indicated that the ethyl acetate fraction of *A. sinensis*, including Z-ligustilide (**3**, Figure 1) and ferulic acid (**38**, Figure 3), can effectively inhibit the IL-1β-induced phosphorylation of ERK1/2, p38 and JNK in the MAPK signaling pathway, as well as the activation of the NF-κB pathway. These regulatory effects further suppress the abnormal proliferation of rheumatoid arthritis synovial fibroblasts and the overproduction of pro-inflammatory mediators and tissue-destructive enzymes including COX-2, PGE_2_, MMP-1, and MMP-3, thereby highlighting the potential of this fraction as a novel therapeutic agent for RA [115]. The regulatory effects of the active components of *A. sinensis* on the bone and joint through cytokines are shown in Table 9.

**Figure 11:**
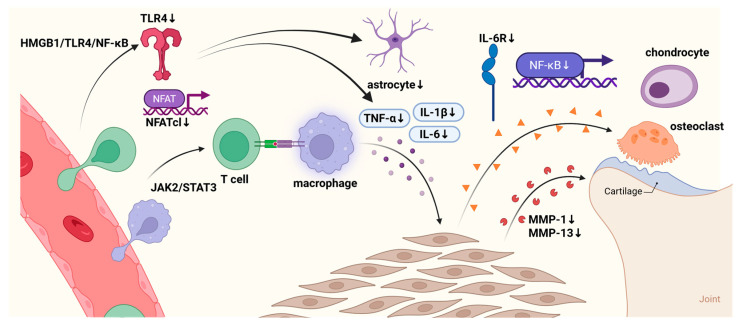
Regulatory effect on bone cells through the immune pathway. Created in BioRender. Li, T. (2026) https://BioRender.com/n26j6lp (accessed on 24 March 2026). (↓ means decrease).

Studies have demonstrated that macrophages, lymphocytes and their associated cytokines are involved in the pathogenesis and progression of osteoarthritis and synovitis [119]. Additionally, ligustilide, ferulic acid and ASP exert anti-inflammatory and analgesic effects, as well as protect chondrocytes and modulate osteoclast activity [120]. Qian et al. [116] demonstrated that ligustilide alleviates inflammatory pain by downregulating TLR4 expression in astrocytes and suppressing the production of NF-κB-mediated chemokines [121]. Wang et al. [117] found that ligustilide can mitigate osteoclast dysfunction by inhibiting the expression of key osteoclastogenic factors, including tumor necrosis factor, IL-6R protein and receptor activator of NF-κB (RANK) mRNA. This effect extended to the downregulation of osteoclast-specific genes dendritic cell-specific transmembrane protein (DC-STAMP) and nuclear factor of activated T cells cytoplasmic 1 (NFATc1), alongside suppression of the NF-κB/ERK/p38/immunoreceptor tyrosine-based activation motif (ITAM) signaling pathway. In addition, LIGc (**8**, Figure 1)—a derivative of (Z)-ligustilide in *A. sinensis*—was found to inhibit the expression of COX-2, PGE_2_ and TNF-α, as well as the protein levels of caspase-3, TLR4, Bcl-2-associated X protein (Bax) and NF-κB p65, while upregulating the anti-apoptotic protein Bcl-2, potentially via the high mobility group box 1 (HMGB1)/TLR4/NF-κB signaling axis [118]. Chen et al. [86] found that ferulic acid (**38**, Figure 3) can reduce hydrogen peroxide-induced expression of IL-1β, TNF-α, MMP-1 and MMP-13, while upregulating *SOX9* gene expression, suggesting its therapeutic potential in osteoarthritis and warranting further research. A schematic diagram illustrating the analgesic effects of active components of *A. sinensis* via the modulation of immune cells and cytokines is presented in Figure 11.

The pathological essence of osteoarthritis is the imbalance of “immune–bone” coupling: aberrant activation of synovial immune cells releases inflammatory factors, which drives osteoclast differentiation and chondrocyte apoptosis, with targets such as STAT3, NF-κB and TLR4 involved in the pathogenesis of osteoarthritis. The active components of *A. sinensis* exert anti-osteoarthritis effects by regulating the immune cell and inflammatory factor network at multiple levels, and their biological effects are centered on core molecular targets including the JAK2/STAT3, MAPK, and HMGB1/TLR4/NF-κB signaling pathways. At the cellular level, ASP inhibits the proliferation, migration, and invasion of FLS and promotes their apoptosis; ligustilide and its derivative LIGc target and inhibit osteoclast differentiation and synovial macrophage activation; ferulic acid suppresses the inflammatory response of chondrocytes and the expression of matrix metalloproteinases. At the molecular level, the above effects are mediated by multiple signaling pathways including JAK2/STAT3, MAPK, NF-κB and HMGB1/TLR4/NF-κB. These components downregulate inflammatory and matrix-degrading mediators such as IL-1β, IL-6, TNF-α, COX-2, PGE_2_, MMP-1/3/13, and upregulate the anti-apoptotic protein Bcl-2 and SOX9, a (a key transcription factor for chondrogenic differentiation), thus forming a coordinated multi-target intervention network that ultimately restores the balance of the “immune–bone” coupling system.

However, current research still has notable limitations. First, the mechanistic depth is insufficient: most studies only stay at the detection of pathway phosphorylation, lacking the identification of direct molecular targets. Second, there is a lack of a holistic and dynamic perspective: most existing studies are static observations that cannot reflect the dynamic evolution of osteoarthritis pathology, and the synergistic effects between various active components have not been systematically evaluated. Third, translational research lags behind: most existing animal models are based on acute intervention, the therapeutic efficacy on advanced osteoarthritis is unknown, and there is a large gap between the administration regimens and clinical practice.

Future research should focus on the following priorities: identifying the direct molecular targets of active components using lineage-specific gene knockout models and osteoclast–immune cell co-culture systems; establishing animal models of different pathological stages to evaluate the stage-specific applicability of the components; dissecting the dynamic changes of the “immune–bone” regulatory network via single-cell transcriptomic analysis; exploring the optimal compatibility of multi-component synergistic intervention; and conducting clinical-aligned pharmacodynamic and pharmacokinetic studies. Collectively, these efforts will not only deepen our understanding of the anti-osteoarthritis mechanisms of *A. sinensis* active components but also lay a robust scientific foundation for the development of targeted, mechanism-based interventions for inflammatory bone disorders.

### 6.4. Anti-Aging by Regulating Cytokines

Accumulating evidence has demonstrated that immune dysregulation is a critical driver of pathological aging. Chronic inflammatory activation and immunosenescence collectively accelerate the aging process and promote the development of age-related diseases [122,123]. Relevant studies have shown that the active components of *A. sinensis* can delay the aging of multiple organs by regulating inflammatory factors. Lu et al. [124] demonstrated that fermented *A. sinensis* can significantly suppress hepatic levels of pro-inflammatory cytokines, including IL-6, TNF-α, IL-1β, concurrently ameliorating aged related liver tissue damage in mice in the liver, with this effect mediated via the Nrf2 pathway/target. Additionally, ASP can regulate endocrine function; it promotes the phosphorylation level of AKT and activates the AKT/FOXO3 signaling pathway, thereby ameliorating immune-mediated premature ovarian failure in mouse models [125]. Mo et al. [126] found that ligustilide can not only enhance the weight of immune organs such as thymus, but also attenuate aging-induced liver and kidney damage by downregulating the expression of inflammatory cytokines including iNOS, COX-2, IκB, etc. Additionally, Zhu et al. [127] found that ligustilide isolated from *A. sinensis* can ameliorates aging-related cognitive impairment by modulating the IL-1β/NF-κB/Bcl-2 signaling axis. In the context of brain aging, ASP was found to downregulate IL-1b, IL-6, TNF-a, and ROS levels, and suppress the expression of senescence-associated genes *p53* and *p21* in neural stem cells, thereby attenuating cellular senescence to delay the aging process [128]. As for the aging of hematopoietic function, ASP can effectively inhibit TNF-α, IL-1, IL-6, promote Runx2 expression, and simultaneously reduce the expression of peroxisome proliferator-activated receptor γ (PPARγ) in bone marrow-derived mesenchymal stem cells (BMSCs) to improve hematopoietic function [129]. The effects of active components from *A. sinensis* on aging through immune system are shown in Table 10.

In summary, the active components of *A. sinensis* exert multi-organ anti-aging effects via regulating the immune–inflammatory network. ASP and ligustilide are the core active components, which regulate the progression of brain aging and premature ovarian insufficiency, intervene in stem cell senescence and multi-organ inflammation. Ligustilide ameliorates aging-related liver and kidney injury, while components from fermented *A. sinensis* exert antioxidant and anti-inflammatory effects via activating the Nrf2 pathway. These effects are mediated by downregulating core inflammatory mediators including IL-1β, IL-6 and TNF-α, and regulating key pathways such as AKT/FOXO3, p53/p21, NF-κB and Nrf2.

However, the underlying mechanism connections behind these phenomena are not yet complete. Currently, it is not clear whether the anti-aging effect of the effective components of *A. sinensis* is achieved by directly reducing the oxidative stress of immune cells. Particularly crucially, the triangular relationship between the effective components of *A. sinensis*, oxidative stress, and specific immune responses has not been systematically studied. For instance, whether T lymphocytes, macrophages, etc. are sensitive to the oxidative–reductive regulation mediated by *A. sinensis*, and whether these effects can be transformed into improved immune surveillance function or delayed immune aging, there are still no clear answers.

Therefore, future research should prioritize focusing on systematically characterizing the effects of *A. sinensis* effective components on the structural and functional integrity of spleen cells and thymus cells in aging models; secondly, using genetic and pharmacological methods, to analyze whether the protective effect on immune cells is achieved by inhibiting the oxidative stress pathway; finally, to clarify the interaction between the oxidative–reductive signals regulated by *A. sinensis* effective components and specific immune responses. Filling these gaps will not only provide a mechanism basis for the immune protective effect of *A. sinensis* in the aging process but also contribute new insights to the in-depth understanding of the oxidative–reductive–immune interaction during aging.

### 6.5. Reducing Intestinal Inflammation by Regulating Inflammatory Factors

As shown in Table 11, the active components of *A. sinensis* can alleviate inflammatory bowel disease (IBD) by regulating inflammatory factors. Studies have confirmed that polysaccharides extracted from the aerial parts of *A. sinensis* can ameliorate inflammatory manifestations in colitis by inhibiting the TLR4/myeloid differentiation primary response gene 88 (MyD88)/NF-κB signaling pathway [130]. In a rat model of immune-mediated colitis, ASP was found to significantly reduce NO production, MPO activity, and the levels of TNF-α and IL-2 [131]. Cho et al. [132] demonstrated that the anti-inflammatory effects of ASP may be mediated by inhibiting neutrophil infiltration into the gastrointestinal mucosa, thereby suppressing gastric mucosal damage. Mechanistically, ASP has been shown to significantly inhibit the release of IL-6, IL-1β, and TNF-α from immune cells, which contributes to the amelioration of ulcerative colitis [133].

The core pathogenesis of IBD lies in the imbalance of the “gut microbiota–immunity–barrier” axis, where dysbiosis triggers a vicious cycle of TLR4 activation, inflammation, and intestinal barrier disruption. ASP, the core anti-IBD bioactive component of *A. sinensis*, exert therapeutic effects via regulating the intestinal immune–inflammatory network. ASP alleviates colonic injury, reduces MPO activity and neutrophil infiltration, and suppresses the TLR4/MyD88/NF-κB pathway to downregulate pro-inflammatory cytokines (IL-1β, IL-6, TNF-α) and inflammatory mediators (NO, PGE_2_), thereby mitigating colitis-related inflammatory damage. It exerts dual therapeutic effects via direct TLR4 signaling inhibition and gut microbiota/metabolite modulation, reflecting its holistic intestinal microecology regulatory advantage.

However, current research has four core limitations. First, the research on the material basis is severely unbalanced: almost all relevant studies focus on ASP, while the anti-IBD efficacy and mechanisms of other active components such as ligustilide and ferulic acid are nearly unexplored. Second, the mechanistic depth is insufficient: the direct interaction between ASP and TLR4 lacks verification, and the direct target of ASP in regulating the pathway remains unclear. Third, there are critical research gaps: microbiota studies mostly stay at phenotypic description at the phylum and genus levels, failing to clarify the causal relationship between microbiota changes and anti-inflammatory effects.

Therefore, future research should focus on the following core priorities: verifying the direct binding between ASP and TLR4; dissecting the key microbiota and metabolites regulated by ASP via integrated multi-omics, and clarifying the causal role of gut microbiota in mediating the therapeutic effects through fecal microbiota transplantation; conducting research on the anti-IBD effects of non-polysaccharide components and exploring the synergistic effects of multiple components, to provide a scientific basis for the clinical application of *A. sinensis* in IBD treatment.

### 6.6. Other Pharmacological Effects

In addition, as shown in Table 12, animal studies have demonstrated that *A. sinensis* exerts anti-fibrotic effects across multiple disease models (epidural, pulmonary, and interstitial lung fibrosis) by downregulating inflammatory and pro-fibrotic mediators including IL-6, TGF-β1, and NF-κB. In addition, the active components of *A. sinensis* can exert metabolic regulatory activities by modulating immune cells and immune-active substances. In fibrosis research, Zhang et al. [134] found that *A. sinensis* alleviated post-laminectomy epidural fibrosis by downregulating factors such as IL-6. Han et al. [135] demonstrated that *A. sinensis* inhibited radiation-induced pulmonary fibrosis by suppressing Tgfb1 expression. Furthermore, *A. sinensis* also ameliorated interstitial lung disease by reducing NF-κB activity and decreasing *TGF-β* mRNA expression in alveolar macrophages [136].

In a rat model of asthma, researchers demonstrated that *A. sinensis* volatile oil can upregulate forkhead box protein P3 (Foxp3) expression and suppress IL-10 expression, thereby exerting anti-asthmatic effects by enhancing Treg cell-mediated immune function [137]. Song et al. [138] found that ASP can inhibit myocardial fibrosis and cardiomyocyte apoptosis in a rat model of hypertensive heart disease. The proposed mechanism involves the downregulation of profibrotic and pro-apoptotic factors—including TGF-β1, Bax, cleaved caspase-9 and cleaved caspase-3—and the upregulation of the anti-apoptotic protein Bcl-2, thereby attenuating the progression of cardiac pathology. In type 2 diabetes, immune cell dysregulation contributes to the aberrant secretion of pro-inflammatory cytokines such as TNF-α, which promotes insulin resistance (IR). Treatment with *A. sinensis* polysaccharides has been shown to reduce the expression of these pro-inflammatory mediators [139].

**Figure 12:**
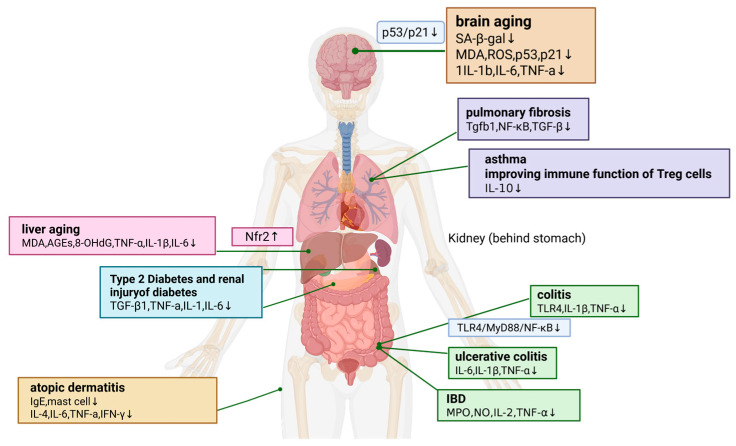
Regulatory effect on diseases throughout the body through the immune path way. Created in BioRender. Li, T. (2026) https://BioRender.com/jtga80b (accessed on 24 March 2026). (↑ means increase; ↓ means decrease).

In diabetic renal injury, innate immunity plays a critical role in disease pathogenesis and progression. Arabinoglucan, isolated from *A. sinensis*, was found to not only suppress the excessive proliferation of glomerular mesangial cells (GMCs) but also downregulate pro-inflammatory mediators including TNF-α, IL-1, IL-6 and TGF-β1 [140]. At the same time, Wang et al. found that the aqueous extract of *A. sinensis* could attenuate endotoxin-induced HMGB1 release in rat models of endotoxemia and sepsis, which may be associated with the interference of HMGB1 cytoplasmic translocation in macrophages [141]. The mechanistic roles of active components of *A. sinensis* in cytokine modulation across various systemic diseases are presented in Figure 12.

In summary, beyond the classical immune-mediated diseases described above, the core bioactive constituents of *A. sinensis* (polysaccharides, volatile oils and their derivatives) exert a wide spectrum of pharmacological effects via targeting the immune regulatory network, including anti-fibrotic, anti-diabetic, renoprotective, hair-regenerative and anti-dermatitic activities. Their common mechanism lies in regulating the function of macrophages, Tregs and other immune cells, and modulating core inflammatory mediators (TNF-α, IL-6, TGF-β1, etc.) through key pathways, including NF-κB, MAPK, and TGF-β1/Smad, to achieve multi-target intervention.

However, the current evidence base in this area exhibits significant heterogeneity that warrants critical consideration. Notable inconsistencies in the studied *A. sinensis* components across disease models hinder cross-disease comparisons: crude extracts (with undefined active ingredients) dominate fibrosis research, while asthma, cardiac/metabolic disease, renal injury and endotoxemia studies focus on volatile oils, ASP, arabinoglucan and aqueous extracts, respectively. Furthermore, the depth of mechanistic validation varies considerably across studies, with many findings remaining at a descriptive level. It remains unclear whether this pattern arises from disease-specific component suitability or researchers’ selection bias—a question that future studies should address through systematic, head-to-head comparisons of different *A. sinensis* constituents in standardized disease models. Such efforts will help distinguish true pharmacological specificity from artifactual research fragmentation, ultimately providing a more coherent foundation for understanding the full therapeutic scope of *A. sinensis* immunomodulation.

## 7. Safety of Active Components in *Angelica sinensis*

Researchers conducted research on the safety of Dang Gui compound and Dang Gui preparations. Zhao et al. [142] found that the combination of Dang Gui Bu Xue Decoction with conventional Western medicine may be more effective than Western medicine alone in the treatment of renal anemia. Notably, when the ratio of Huang Qi to Dang Gui is 5:1, significant improvements in red blood cell (RBC) count, hematocrit (HCT), and overall clinical efficacy are observed, with no adverse reactions reported in the experimental group. Yang et al. [143] found that *A. sinensis* injection has no toxic effect on the survival of chicken embryos.

Multiple pharmacological studies have shown that polysaccharides extracted from *A. sinensis* have hepatoprotective effects and can effectively alleviate liver damage and toxicity. The cell assay of ASR-CD in RAW264.7 cells showed no cytotoxicity, indicating good biocompatibility [144]. Yue et al. [145] proved that *A. sinensis* is not a stimulator of breast cancer through in vivo and in vitro experiments, but *A. sinensis* should still be used cautiously in patients with estrogen receptor positive breast cancer.

In the safety study of *A. sinensis* essential oil, Ma et al. [146] topically applied the essential oil at concentrations of 5%, 3%, and 1% to a dehydrotestosterone-induced androgenetic alopecia mouse model. Safety was evaluated via acute oral, dermal, and ocular irritation toxicity tests, with results showing no evidence of acute toxicity. In addition, in the safety assessment of LIGc (**8**, Figure 1), no significant changes in organ weights were observed in treated mice, and histopathological examination revealed no tissue or organ abnormalities, indicating good overall health [147]. In a study on the material basis and molecular mechanisms of the blood-activating effects of eight constituents from *A. sinensis*, (Z)-ligustilide (**3**, Figure 1), senkyunolide A (**7**, Figure 1), levistolide A (**32**, Figure 2), ferulic acid (**38**, Figure 3), isoquercitrin (**104**, Figure 6), imperatorin (**116**, Figure 7), osthole (**118**, Figure 7) and scopolamine (**122**, Figure 7), were found to exhibit low central neurotoxicity. These safety-related predictions were performed using QikProp (Schrödinger, LLC), a computational chemistry tool specialized in evaluating ADMET (Absorption, Distribution, Metabolism, Excretion, Toxicity) properties of small molecules. All compounds except imperatorin showed QikProp log (QPlg) human ether-a-go-go-related gene (hERG) values within the acceptable range, indicating low cardiotoxicity risk. Predicted hepatotoxicity via the software’s built-in toxicological prediction module suggested no hepatic toxicity for all eight anticoagulant constituents. Additionally, the median lethal dose (LD_50_) of levistolide A (**32**, Figure 2) was predicted as 100 mg·kg^−1^ (class III) using admetSAR (a free online ADMET prediction platform), implying potential safety risks, whereas the other seven components were classified as class IV or V with favorable safety profiles [53].

## 8. Regulatory and Pharmacopeial Perspective on *Angelica sinensis*: Quality Control and Immunological Implications

### 8.1. The Current Status of Quality Control

The 2020 edition of Pharmacopoeia of the People’s Republic of China (Volume I) (Chp) [148] has established a comprehensive quality control system for *A. sinensis* (encompassing raw and processed products) that includes origin identification, content determination and safety evaluation. With ferulic acid and volatile oil as core quantitative indicators, this system provides a chemical reference for ensuring the reproducibility of immunomodulatory effect studies on *A. sinensis*. Notably, these pharmacopeial standards offer a robust scientific benchmark for the quality control of Chinese medicinal materials and are critical to the reproducibility of pharmacological studies—particularly those involving complex immunomodulatory effects. For international researchers, understanding and adopting such standards (e.g., a ferulic acid content of no less than 0.050%) is fundamental to generating comparable and translatable research data. The characteristic identification, content determination, safety control, and relevant standards for wine-processed *A. sinensis* are summarized in Table 13.

### 8.2. The Impact of Quality Control on Immunology Research

The immunomodulatory effects of *A. sinensis* are mediated by its core active components, including ferulic acid (38, Figure 3), ASP, and (Z)-ligustilide (3, Figure 1). The quality control system for *A. sinensis* established in the *Chinese Pharmacopoeia (2020 Edition)* provides a fundamental quality guarantee for its clinical application.

To date, in studies on the spectrum–effect relationship of *A. sinensis*, researchers have identified multiple effective components with antioxidant activity, such as (Z)-ligustilide (3, Figure 1), (E)-ligustilide (4, Figure 1), and butylphthalide [149]. In a rat model of blood deficiency, ferulic acid (38, Figure 3) has been recognized as the key marker component for tonifying blood and qi, and it can restore hematopoiesis by restoring thymus weight [150]. Meanwhile, in a mouse model of qi deficiency, Yang et al. found that ligustilide, and butylphthalide can also restore immune organ weights and enhance the phagocytic function of mononuclear macrophages. These findings provide a scientific basis for exploring the immunomodulatory mechanisms of *A. sinensis* active components via immune-mediated pathways.

However, quality control research of *A. sinensis* related to immunomodulation still has considerable room for improvement. First, there is no exclusive quality control standard for ASP—the most immunologically active component—with current standards relying on indirect control via extract content. Despite recent clarification of ASP’s monosaccharide composition and partial glycosidic linkages using HPGPC and NMR, structural differences (branching degree, modifications) across producing areas and medicinal parts lead to poor reproducibility of its immunopharmacological effects. Second, the *Chinese Pharmacopoeia* only uses total volatile oil as an indicator, failing to reflect the (Z)/(E)-ligustilide isomer ratio and thus unable to accurately evaluate stereoisomeric differences in immunomodulatory and anti-inflammatory activities. Third, existing standards do not differentiate quality control among *A. sinensis* head, body, and tail; the tail, a key immunomodulatory part with higher ferulic acid (38, Figure 3) and polysaccharide contents, when mixed with other parts, causes active ingredient imbalance and weakened immunomodulatory stability.

Future research should integrate “component–structure–efficacy” correlations and advances in structural analysis/rapid detection technologies to incorporate ASP-specific indicators, (Z)/(E)-ligustilide ratio, and tail-specific quality control standards into the pharmacopeia. Establishing a quantitative relationship between processing technology and immunologically active ingredients will upgrade the quality control system from “qualified quality” to “stable efficacy”, providing a solid legal basis for the precise clinical application of *A. sinensis* in immunomodulation.

### 8.3. International Perspectives

Although *A. sinensis* has well-established pharmacopeial standards and a long clinical history in China, it has not yet been granted an EU herbal monograph by the EMA/HMPC. In a 2013 public statement, the EMA/HMPC cited its failure to meet EU “well-established/traditional use” criteria, owing to insufficient efficacy and safety evidence, the lack of a required 30-year medicinal use history (15 years within the EU), and preliminary toxicological concerns over its phytochemical constituents.

Nevertheless, this assessment is over a decade old, and the extensive new scientific evidence summarized in this review provides a robust basis for re-evaluating these conclusions. Mechanistic and preclinical studies have clarified the immunomodulatory effects of key active components, such as, ASP, (Z)-ligustilide (**3**, Figure 1) via specific signaling pathways (e.g., NF-κB, JAK/STAT)—extending beyond traditional uses and providing a biological rationale for indications such as anemia and inflammatory disorders. Furthermore, updated safety data, such as low cytotoxicity and hepatoprotective effects of its polysaccharides, address earlier toxicological uncertainties. Its long, well-documented use in the *Chinese Pharmacopoeia*, combined with modern scientific validation, further supports the feasibility and safety of *A. sinensis* under defined conditions. While the lack of long-term European use evidence remains a regulatory challenge, its consistent TCM application is strongly backed by advanced pharmacological and toxicological research.

This robust modern pharmacological and toxicological data thus provides a compelling rationale for re-evaluating the EU’s regulatory position on *A. sinensis*. To advance this, future research should prioritize high-quality randomized controlled trials in EU populations and targeted toxicological risk assessments of specific constituents. Aligning the reliability of traditional use, modern scientific validation, and regional regulatory requirements is key to facilitating the responsible global application of *A. sinensis* in complementary and alternative medicine.

## 9. Conclusions and Future Prospective

Based on the preceding discussion, this review systematically elaborates the immunomodulatory effects and underlying mechanisms of *A. sinensis* and its core bioactive constituents, including ASP, phthalides (represented by (Z)-ligustilide), and phenolic acids (represented by ferulic acid). The immunomodulatory effects of *A. sinensis* show clear constituent specificity: ASP acts primarily through TLR4, STAT, and PI3K pathways, establishing it as the core component with stable activity across all disease models; phthalides, represented by (Z)-ligustilide (**3**, Figure 1), mainly exert anti-inflammatory organ-protective effects via NF-κB, MAPK signaling pathways; and phenolic acids, represented by ferulic acid (**38**, Figure 3), focus on antioxidant and metabolic regulation through Th2 and Caspase 3 pathways. Accumulating evidence confirms that *A. sinensis* and its active components exert effects via a tripartite mode of “anti-inflammation–antioxidation–immune homeostasis regulation”: they restore immune organ structure and function, regulate immune cell activation and polarization, modulate inflammatory mediator secretion through core pathways including NF-κB, PI3K/Akt, and JAK/STAT, and exhibit therapeutic potential in multiple immune-mediated diseases, such as malignant tumors, anemia, osteoarthritis, inflammatory bowel disease, fibrotic diseases, and aging-related disorders. Compared with single-target chemical immunomodulators, the multi-component, multi-target holistic regulatory feature of *A. sinensis* enables bidirectional immune homeostasis regulation rather than simple immune suppression or activation, highlighting its unique advantages in treating chronic immune-mediated diseases.

These findings lay a solid theoretical foundation for clarifying the immunomodulatory properties of *A. sinensis*, but three core bottlenecks remain to be addressed. First, research on the material basis is unbalanced and fragmented. Current studies are dominated by ASP, while non-polysaccharide components are seriously understudied, lacking clarity on the synergistic effects of multiple components; the disease-specific research pattern hinders cross-disease comparisons, and it remains unclear whether efficacy differences stem from component specificity or research bias. Second, the mechanistic depth is insufficient: most studies stay at the phenotypic description of “component–effect–pathway”, mostly providing correlative rather than causal evidence, and the direct molecular targets of most active components have not been systematically verified. Third, the translational value is limited: most studies adopt acute models and preventive administration, which are disconnected from the chronic pathological process in clinical settings; non-standardized preparation quality control leads to poor result reproducibility, and there is a lack of high-quality pharmacokinetic studies and randomized controlled trials to support clinical application.

Therefore, future research should focus on clarifying core active monomers via bioactivity-guided isolation, systematically exploring the synergistic effects of multiple components, taking direct target identification and causal mechanism verification as the core to clarify the underlying mechanism of active components, integrating multi-omics and in vivo models to dissect the regulatory effect of *A. sinensis* on the “microbiota–immune–metabolism” axis, exploring the common cross-disease regulatory network, establishing a standardized quality control system for *A. sinensis* preparations, conducting standardized preclinical and clinical trials, and promoting its transformation from traditional empirical medicine to evidence-based immunotherapy.

## Figures and Tables

**Table 1:** Traditional and Modern Clinical Applications of *A. sinensis*.

**Traditional Uses**				
**Source of the literature**		**Functions and Indications**		**Ref.**
*Shennong’s Herbal Classic of Materia Medica*		cough with dyspnea, alternate chills and fever, postpartum hemorrhage, infertility, traumatic injuries by metal, ulcerative sores		[11]
*The Appraisal of Medicinals*		headaches (+Chuan Xiong *Ligusticum chuanxiong*), tonifying blood (+Huang Qi *Astragalus membranaceus*, Ren Shen *Panax ginseng*), activating blood, resolving stasis (+Qian Niu *Ipomoea nil*, Da Huang *Rheum rhabarbarum*)		[12]
**Modern TCM Clinical Uses**				
**Disease**	**Formulations/Preparations**		**Clinical Effects**	**Ref.**
advanced cervical cancer	Modified Dang Gui Shao Yao San + paclitaxel+cisplatin		↑ CD3^+^, CD4^+^, CD4^+^/CD8^+^ ratio, immunoglobulins ↓ IL-6, IL-1β, TNF-α	[13]
breast cancer (surgery or postoperative chemotherapy)	Dang Gui Shao Yao San		↓ upper limb lymphedema, myelosuppression	[14]
advanced gastric cancer	Yi Qi Fu Zheng Jie Du Decoction + Compound *A. sinensis* injection		↓ CEA, CA125, TPS ↑ CD3^+^, CD4^+^, CD4^+^/CD8^+^ ratio	[15]
chronic anemia	Dang Gui Bu Xue Decoction		↑ CD4^+^, CD4^+^/CD8^+^ ratio ↑ hemoglobin, RBC	[16]
preterm infants with anemia			↑ Hb concentration, RBC, HCT	[17]
tumor-related anemia			↑ Hb, RBC, HCT, CD4^+^/CD8^+^ ratio	[18]
knee osteoarthritis	Dang Gui Nian Tong Decoction + moxibustion		↓ joint swelling and pain, IL-1β, TNF-α	[19]
acute gouty arthritis	Dang Gui Shao Yao San		↓ serum uric acid, C-reactive protein	[20]
knee osteoarthritis	Modified Dang Gui Shao Yao San		↓ osteoarthritis index, lysholm scores	[21]
Alzheimer’s disease	Dang Gui Shao Yao San + acupuncture		↓ restlessness, agitation, hyperactivity, insomnia, delusions, depression	[22]
	Dang Gui Shao Yao San + carbamazepine		↑ VEGF, miR-132, cognitive function ↓ IL-1β, NF-κB, miR-146a	[23]
mild cognitive impairment	Bu Yang Huan Wu Decoction		↓ progression ↑ cognitive performance	[24]
primary dysmenorrhea	Compound *A. sinensis* injection (acupoint injection)		↓ VAS	[25]
ulcerative colitis	*A. sinensis* injection		↑ GMP-140, TXB(2)	[26]
diabetic nephropathy	Dang Gui Si Ni San + Si Miao Pills		↓ IL-6, TNF-α, MDA ↑ kidney function	[27]
diabetic peripheral neuropathy	Modified Dang Gui Si Ni San + acupuncture + topical herbal washes		↓ whole blood viscosity ↑ nerve conduction velocity	[28]

Abbreviations: TCM, traditional Chinese medicine; CEA, carcinoembryonic antigen; CA125, carbohydrate antigen 125; TPS, tissue polypeptide specific antigen; CD3^+^, cluster of differentiation 3-positive T lymphocytes; CD4^+^, cluster of differentiation 4-positive T lymphocytes; CD8^+^, cluster of differentiation 8-positive T lymphocytes; IL-6, interleukin-6; IL-1β, interleukin-1β; TNF-α, tumor necrosis factor-α; RBC, red blood cell; Hb, hemoglobin; HCT, hematocrit; VEGF, vascular endothelial growth factor; VAS, visual analog scale; GMP-140, granule membrane protein 140; TXB(2), thromboxane B2; MDA, malondialdehyde; miR-132, microRNA-132; miR-146a, microRNA-146a; NF-κB, nuclear factor kappa-B. ↑ means increase; ↓ means decrease.

**Table 2:** Methods for the Identification of Chemical Compounds from *A. sinensis*.

Chemical Compound	Identification Methods	Ref.
APS-1cI and APS-1cII	GC–MS (Agilent 6890N/5973N, Santa Clara, CA, USA), NMR (Bruker Avance 500 MHz, Billerica, MA, USA)	[29]
Unspecified polysaccharides	HPLC, (Waters 2695/2996, Milford, MA, USA), HPSEC (Waters 2695/2414, Milford, MA, USA)	[30]
APS-1a, APS-3a	NMR (Bruker AM-400, Bruker Corporation, Billerica, MA, USA)	[31]
CAPS_30_, CAPS_50_, CAPS_70_, CAPS_80_	HPGPC (Wyatt HPGPC system, Wyatt Technology Corp., Santa Barbara, CA, USA), GC-MS (Shimadzu GCMS-QP2010 Ultra, Shimadzu Corporation, Kyoto, Japan), FT-IR (Nicolet 6700 FT-IR Spectrophotometer, Thermo Fisher Scientific, Waltham, MA, USA)	[32]
phthalide monomers (**1**–**20**)	NMR (Varian/Bruker, Palo Alto, CA, USA), MS (Waters/Finnigan/Micromass, USA/UK)	[33,35,36,37,38]
phthalein dimers (**21**–**37**)		
phenolic acids and their derivatives (**38**–**43**)	NMR (Bruker AM-400, Billerica, MA, USA), HPLC (Waters 515, Milford, MA, USA), MS (Finnigan MAT 95, San Jose, CA, USA; Esquire 3000plus, Bremen, Germany)	[35,37,39,40]
phenylpropanoid esters (**44**–**45**)		
phenylpropanoid glycosides (**46**)		
coumarins (**47**–**48**)		
other related compounds (**49**–**50**)		
monoterpenoids (**51**–**63**)	GC–MS (Finnigan Trace Mass, Thermo Fisher Scientific Inc., Waltham, MA, USA)	[45,46]
sesquiterpenoids (**64**–**73**)		
aromatic ketone (**74**)	NMR (Bruker ARX 300/AV 600, Billerica, MA, USA), MS	[37,46,47,48]
phenolic compounds (**75**–**84**)		
phenolic glycosides (**85**–**86**)		
benzoic acid (**87**–**91**)		
aromatic aldehydes (**92**–**95**)		
phthalate esters (**96**–**99**)		
flavonols (**100**–**105**)	NMR (Bruker ARX 300/AV 600, Billerica, MA, USA), HPLC-PDA (Shimadzu SIL-20AC HT/SPD-M20A, Kyoto, Japan), UV–Vis (Unicam HELIOS-α, Cambridge, UK) UHPLC-QE–MS (Thermo Fisher Orbitrap Exploris 120/Vanquish UHPLC, Waltham, MA, USA)	[48,49,50,51]
flavones (**106**–**109**)		
flavanones (**110**–**112**)		
chalcones (**113**–**114**)		
coumarin derivatives (**115**–**118**)	NMR (Bruker ARX 300/AV 600, Billerica, MA, USA), MS	[48,52,53]
alkaloids (**119**–**122**)		

Abbreviations: GC–MS, gas chromatography–mass spectrometry; NMR, nuclear magnetic resonance; HPLC, high-performance liquid chromatography; LC–MS, liquid chromatography–mass spectrometry; UHPLC-QE–MS, ultra-high performance liquid chromatography–Q exactive mass spectrometry; HPGPC, high-performance gel permeation chromatography; HPLC-PDA, high-performance liquid chromatography–photodiode array detector; UV–Vis, ultraviolet–visible spectroscopy; MS, mass spectrometry, FT-IR, Fourier transform infrared spectroscopy.

**Table 3:** Effects of ASP from *A. sinensis* on the immune organs.

No.	Chemical Component	Model	Pathways	Effects	Ref.
1	ASP-1a ASP-3a	In vivo: immune injury mouse model	/	↑ thymus, spleen indices ↑ bone marrow cells	[31]
2	ASP	In vivo: splenic injury mouse model In vitro: splenic cell injury mouse model	PI3K/AKT keap1/Nrf2	↑ spleen weight, peripheral blood leukocytes, lymphocytes, ↑ IL-2, IL-6, IFN-γ	[54]
3	APS-1	In vitro: murine leukemia virus infected mouse model	/	↑ thymus/body mass index ↑ CD4^+^ cells, CD4^+^/CD8^+^ ↓ virus replication	[55]

Abbreviations: ASP, *Angelica sinensis* polysaccharides; APS-1a, *Angelica* polysaccharide-1a; APS-3a, *Angelica* polysaccharide-3a; PI3K/AKT, phosphoinositide 3-kinase/protein kinase B; keap1, Kelch-like ECH-associated protein 1; Nrf2, nuclear factor erythroid 2-related factor 2; IL-2, interleukin-2; IFN-γ, interferon-γ. ↑ means increase; ↓ means decrease.

**Table 5:** Inhibitory effect of active components from *A. sinensis* on immune cells.

No.	Chemical Component	Model	Pathways	Effects	Ref.
1	ferulic acid (**38**, Figure 3)	In vivo: MN mouse model	/	↓ Th2, IgG1, IgG2a ↓ AOPP, SOD, CAT, GPx	[71]
2	ASP	In vitro: RBL-2H3 mast cell model	Gab2/PI3K/Akt Fyn/Syk	↓ mast cells, TNF-α, IL-1, IL-4, IL-6	[72]
3	n-butyl phenolphthalein	In vitro: DC2.4 dendritic cell model BALB/c mouse spleen cell model	NF-κB	↓ DC2.4 dendritic cell activation, IL-6, TNF-α	[73]
4	ASP	In vitro: mouse splenic cell model	/	↓ splenic cell, CD4^+^T cell ↑ CD19^+^B cell	[74]

Abbreviations: Th2, T helper 2 cells; IgG1, immunoglobulin G1; IgG2a, immunoglobulin G2a; AOPP, advanced oxidation protein products; SOD, superoxide dismutase; CAT, catalase; GPx, glutathione peroxidase; IL-1, interleukin-1; IL-4, interleukin-4; Gab2, GRB2-associated binding protein 2; Fyn/Syk, proto-oncogene tyrosine–protein kinase/spleen tyrosine kinase; DC2.4, murine dendritic cell line DC2.4; CD19^+^B cells, cluster of differentiation 19-positive B lymphocytes. ↑ means increase; ↓ means decrease.

**Table 6:** Effects of main chemical components from *A. sinensis* on the immune active substances.

No.	Chemical Component	Model	Pathways	Effects	Ref.
1	ASP	In vivo: liver injury mouse model	Caspase-8, JNK, IL-6/STAT3, NF-κB	↓ IL-2, IL-6, TNF-α, IFN-γ ↓ ROS, MDA	[74]
2	ASP	In vivo: RA rat model	Janus kinase/signal transducers, IL-6/JAK2/STAT3	↓ TNF-α, IL-6	[76]
3	ASP	In vivo: acute lung injury mouse model	NLRP3, NF-κB	↓ neutrophils, macrophages ↓ IL-6, IL-1β, IL-18, MPO, TNF-α	[77]
4	volatile oil of *A. sinensis*	In vivo: acute inflammation rat model	/	↓ WBC, NE%, IL-1β, IL-6, NO, TNF-α ↑ IL-10	[78]
5	volatile oil of *A. sinensis*	In vivo: acute inflammation rat model	/	↓ PGE_2_, TNF-α	[79]
6	volatile oil of *A. sinensis*	In vivo: acute inflammation rat model	histidine, tryptophan metabolism, steroid hormone biosynthesis, fatty acid metabolism, energy metabolism	↓ TNF-α, IL-6, IL-1β, NO	[80]
7	volatile oil of *A. sinensis*	In vivo: ear edema mouse model	/	↓ TNF-α, COX-2, IL-6	[81]
8	ASP	In vivo: chronic liver fibrosis mouse model In vitro: mouse hepatic stellate cell model	IL-22/STAT3	↑ IL-22 ↓ TNF-α, IL-6	[82]
9	ferulic acid (**38**, Figure 3), ligustilide	In vivo: endotoxin shock mouse model In vitro: mouse RAW 264.7 macrophages model	NF-κB	↓ NF-κB, TNF-α, IL-6, MIP-2, NO	[83]
10	glabralactone	In vivo: paw edema rat model In vitro: rat RAW 264.7 macrophages model	IRF-3, NF-κB	↓ NO, iNOS, TNF-α, IL-1β, miR-155 mRNA	[84]
11	ligustilide	In vitro: RAW264.7 mouse macrophages model	MAPKs/IKK	↓ NO, PGE_2_, TNF-α, iNOS, AP-1, NF-κB	[85]
12	ferulic acid (**38**, Figure 3)	In vitro: porcine knee chondrocyte model	/	↓ IL-1β, TNF-α, MMP-1 ↑ *SOX9*	[86]

Abbreviations: NLRP3, NOD-like receptor family pyrin domain containing 3; JAK2/STAT3, janus kinase 2/signal transducer and activator of transcription 3; MPO, myeloperoxidase; WBC, white blood cell; NE%, neutrophil percentage; PGE_2_, prostaglandin E2; IRF-3, interferon regulatory factor 3; TRIF, TIR-domain-containing adaptor protein inducing IFN-β; MAPK, mitogen-activated protein kinase; AP-1, activator protein-1; IKK, inhibitor of nuclear factor kappa-B kinase; IL-22, interleukin-22; MIP-2, macrophage inflammatory protein-2; SOX9, SRY-box transcription factor 9; MMP-1/13, matrix metalloproteinase-1/13. ↑ means increase; ↓ means decrease.

**Table 7:** Inhibitory effect of *A. sinensis* on tumor cells through the immune system.

No.	Chemical Component	Model	Pathways	Function	Ref.
1	*A. sinensis* phenolic acid and phthalein components	In vivo: colorectal cancer mouse model	/	↓ i NOS, COX-2 ↓ cell proliferation activity, DNA damage	[87]
2	AASP	In vivo: H22 liver cancer cell, tumor-bearing mouse model	/	↑ splenocytes, peritoneal macrophages, NK cells ↑ TNF-α, IL-2, IFN-γ	[93]
3	ASP	In vivo: nude mouse lung metastasis A549 cells model In vitro: lung adenocarcinoma A549 cells	/	↓ MMP-2, MMP-9, TGF-β1	[88]
4	ASP	In vivo: tumor implants mouse model In vitro: human normal liver cell line (L-02) model, human hepatocellular carcinoma cell line (HepG2) model	JAK/STAT, BMP-SD	↓ IL-6, JAK2, p-STAT3, p-SMAD1/5/8	[89]
5	AP-0	In vivo: sarcoma S180, ehrlich ascites carcinoma EAC, leukemia L1210 In vitro: human liver cancer cell line HHCC, human embryonic skin fibroblast cell line Fb	/	↓ thymus weight	[94]
6	(Z)-ligustilide (**3**, Figure 1)	In vivo: Balb/C mouse CT26 homologous transplantation tumor In vitro: human colorectal cancer cell line HCT116, human mononuclear cell THP-1 (Type M2), HCT116 + THP-1-M2	/	↓ Ki67, MMP9, CD206	[95]
7	ASP	In vitro: mouse peritoneal macrophages model	/	↑ NO, TNF-α, ROS, iNOS, LSZ ↓ L929 cells	[90]
8	ASP	In vitro: human non-small cell lung cancer A549 cell model, human breast cancer MCF-7 cell model, mouse splenocyte model	/	↑IL-2, Th1/Th2 ↓IL-10	[96]

Abbreviations: MMP-2/9, matrix metalloproteinase-2/9; TGF-β1, transforming growth factor-β1; JAK/STAT, janus kinase/signal transducer and activator of transcription; BMP-SMAD, bone morphogenetic proteins/Sma- and Mad-related proteins; AASP, alkaline-soluble polysaccharide; HHCC, human hepatocellular carcinoma cell line; Fb, human embryonic skin fibroblast cell line; S180, sarcoma S180; EAC, ehrlich ascites carcinoma; L1210, leukemia L1210; CD206, cluster of differentiation 206; Ki67, proliferation marker protein Ki67; L929 cells, murine fibrosarcoma cell line L929; MCF-7, human breast cancer cell line MCF-7; ROS, reactive oxygen species; Th1/Th2, T helper 1 cells/T helper 2 cells. ↑ means increase; ↓ means decrease.

**Table 8:** Improving hematopoietic function of chemical components from *A. sinensis*.

No.	Chemical Component	Model	Pathways	Function	Ref.
1	ASP	In vivo: stress anemia mouse model	/	↑ RBC, hemoglobin, hematocrit, F4/80^+^ VCAM-1^+^ central macrophages	[101]
2	ASP	In vivo: blood deficiency mouse model	/	↑ EPO, G-CSF, IL-3, hematopoiesis ↓ TNF-α	[105]
3	ferulic acid (**38**, Figure 3), (Z)-ligustilide (**3**, Figure 1)	In vivo: strenuous exercise anemia rat model	/	↑ red blood cell, hemoglobin ↓ IL-6	[106]
4	ASP	In vivo: acute hemorrhagic anemia mouse model In vitro: mouse GM colony model	/	↑ CFU-GM, GM-CSF, IL-6, IL-3, hemoglobin	[102]
5	ASP	In vivo: aplastic anemia mouse model In vitro: AA model mice’ nucleated cells in bone marrow model	p38/MAPK	↑ peripheral blood cells, hemoglobin ↑ Treg/Th17, Bcl-2, BMNCs ↓ IL-17	[103]
6	ASP	In vivo: chronic kidney disease anemia In vitro: Hep3B cells under hypoxia/inflammation	/	↓ p-STAT3, p-SMAD1/5/8, DMT1, TfR1 ↓ WBC, TNF-α, IL-1 β, IL-6 ↑ EPO	[109]
7	AAP	In vivo: ACD rat model In vitro: HepG2 cell inflammation model	IL-6/STAT3, BMP/SMAD, IKKs-IκBα	↑ EPO, red blood cells ↓ TNF-α, IL-6, hepcidin protein	[110]
8	ASP	In vitro: human CD34^+^ hematopoietic stem cell model, CD34^+^ cells in bone marrow	/	↑ GM-CSF, IL-3, CD34^+^ cells	[104]

Abbreviations: VCAM-1, vascular cell adhesion molecule-1; EPO, erythropoietin; G-CSF, granulocyte colony-stimulating factor; GM-CSF, granulocyte–macrophage colony-stimulating factor; CFU-GM, colony-forming unit-granulocyte macrophage; AA, aplastic anemia; Treg/Th17, regulatory T cells/T helper 17 cells; Bcl-2, B cell lymphoma/leukemia-2; BMNCs, bone marrow nucleated cells; CKD, chronic kidney disease; DMT1, divalent metal ion transporter 1; TfR1, transferrin receptor 1; ACD, anemia of chronic disease; HepG2, human hepatocellular carcinoma cell line HepG2; BMP/SMAD, bone morphogenetic proteins/Sma- and Mad-related proteins; IKKs-IκBα, IκB kinases-inhibitor of NF-κB α; CD34^+^, cluster of differentiation 34-positive cells. ↑ means increase; ↓ means decrease.

**Table 9:** Anti-osteoarthritis of active components from *A. sinensis* by regulating cytokines.

No.	Chemical Component	Model	Pathways	Function	Ref.
1	ASP	In vivo: arthritis rat model In vitro: fibroblast-like synovial cells model	JAK2/STAT3 MAPK	↓ IL-6, IL-1β, iNOS, FLS cells	[114]
2	ligustilide	In vivo: chronic inflammatory pain In vitro: primary astrocyte inflammation	/	↓ TLR4, TLR4 mRNA	[116]
3	ferulic acid (**38**, Figure 3)	In vitro: osteoarthritis	/	↓ IL-1β, TNF-α, MMP-1, MMP-13 ↑ SOX9	[86]
4	*A. sinensis* ethyl acetate	In vitro: FLS of rheumatoid arthritis	MAPK/NF-κB	↓ NF-κB, COX-2, PGE_2_, MMP-1 MMP-3	[115]
5	ligustilide	In vitro: model of differentiation of bone marrow-derived macrophages into osteoclasts	NF-κB/ERK/p38/ITAM	↓ thermal hyperalgesia, NFATc1, KC, p-NF-κB	[117]
6	LIGc (**8**, Figure 1)	In vitro: inflammation of OA chondrocytes	HMGB1/TLR4/NF-κB	↓ COX-2, PGE_2_, TNF-α, Bax, caspase-3, TLR4, NF-κB p65	[118]

Abbreviations: FLS cells, fibroblast-like synovial cells; LIGc, ligustilide derivative (**8**, Figure 1); OA, osteoarthritis; HMGB1, high mobility group box 1; ERK, extracellular signal-regulated kinase; p38, p38 mitogen-activated protein kinase; ITAM, immunoreceptor tyrosine-based activation motif; NFATc1, nuclear factor of activated T cells c1; KC, keratinocyte chemoattractant; Bax, Bcl-2-associated X protein; caspase-3, cysteine-aspartic acid protease 3; NF-κB p65, nuclear factor kappa-B p65 subunit. ↑ means increase; ↓ means decrease.

**Table 10:** Effects of active components from *A. sinensis* on aging through immune system.

No.	Chemical Component	Model	Pathways	Function	Ref.
1	3-phenyllactic acid, L-5-hydroxytryptophan, paclitaxel, methyl gallate	In vivo: aging mouse model	Nrf2	↓ MDA, TNF-α, IL-1β, IL-6 ↑ Nrf2	[124]
2	ASP	In vivo: premature ovarian failure mouse model	AKT/FOXO3	↑ IL-1β, IL-6, SOD ↓ MDA	[125]
3	ligustilide	In vivo: liver, kidney damage mouse model	/	↑ thymus weight ↓ iNOS, COX-2, IκB	[126]
4	ligustilide	In vivo: aging mouse model	/	↓ IL-1β, NLRP3, NF-κB, Bcl-2	[127]
5	ASP	In vivo: brain tissue aging mouse model In vitro: mouse neural stem cell aging model	p53/p21	↓ MDA, IL-1b, IL-6, TNF-α, ROS ↑ SOD, T-AOC	[128]
6	ASP	In vivo: aging rat model In vitro: BMSCs/HSPCs aging model	/	↓ IL-1β, IL-6, TNF-α	[129]

Abbreviations: FOXO3, forkhead box O3; IκB, inhibitor of nuclear factor kappa-B; p53, tumor protein p53; p21, cyclin-dependent kinase inhibitor 1; T-AOC, total antioxidant capacity; BMSCs, bone marrow mesenchymal stem cells; HSPCs, hematopoietic stem and progenitor cells. ↑ means increase; ↓ means decrease.

**Table 11:** Immunomodulatory effect of ASP on intestinal inflammation.

No.	Chemical Component	Model	Pathways	Function	Ref.
1	ASP-Ag-AP	In vivo: ulcerative colitis mouse model In vitro: IPEC-J2 cell inflammation model	TLR4/MyD88/NF-κB	↓ TLR4, IL-1β, TNF-α	[130]
2	ASP	In vivo: immune colitis rat model	/	↓ NO, MPO, TNF-α, IL-2	[131]
3	ASP	In vivo: gastric damage rat model	/	↓ MPO, PGE_2_	[132]
4	ASP	In vivo: ulcerative colitis mouse model In vitro: Caco-2 cell intestinal barrier damage model	/	↓ IL-6, IL-1β, TNF-α, MPO	[133]

Abbreviations: ASP-Ag-AP, ASP-silver complex with aptamer; IPEC-J2 cells, porcine intestinal epithelial cell line J2; MyD88, myeloid differentiation primary response 88; Caco-2 cells, human colorectal adenocarcinoma cell line Caco-2. ↓ means decrease.

**Table 12:** Immunomodulatory effect of active components from *A. sinensis* on other diseases.

No.	Chemical Component	Model	Pathways	Function	Ref.
1	*A. sinensis* volatile oil	In vivo: asthma rat model	/	↑ Foxp3 ↓ IL-10	[137]
2	ASP	In vivo: hypertensive heart disease rat model	/	↑ TGF-β1, Bax, cleaved caspase-3, cleaved caspase-9, Bcl-2	[138]
3	ASP	In vivo: type 2 diabetes mouse model	/	↓ TNF-α, IL-6	[139]
4	arabinoglucan	In vivo: type 1 diabetes rat model In vitro: renal glomerular mesangial cell injury model	/	↓ GMCs, TGF-β1, TNF-α, IL-1, IL-6	[140]

Abbreviations: Foxp3, forkhead box P3; cleaved caspase-3, cleaved cysteine-aspartic acid protease 3; cleaved caspase-9, cleaved cysteine-aspartic acid protease 9; GMCs, glomerular mesangial cells.↑ means increase; ↓ means decrease.

**Table 13:** The requirements of the Chinese Pharmacopoeia for the quality control of *A. sinensis*.

Category	Pharmacopeial Standards (ChP 2020)
Characteristic Identification	Characteristic chromatogram with seven common peaks, similarity ≥ 0.90
Assay	ferulic acid: ≥0.050% volatile oil: ≥0.4% mL/g
Safety Tests	Moisture ≤ 15.0%, Total ash ≤ 7.0%, Acid-insoluble ash ≤ 2.0% Heavy metals: Pb ≤ 5, Cd ≤ 1, As ≤ 2, Hg ≤ 0.2, Cu ≤ 20 (mg/kg)
Processing (Wine-processed)	Moisture ≤ 10.0%; extractives ≥ 50.0%

Note: These are standardized quality control parameters used to ensure herbal material consistency. Ferulic acid and Volatile oil are key active marker compounds. Total ash measures inorganic impurities, while Acid-insoluble ash indicates siliceous contaminants (e.g., soil). Extractive value reflects the amount of soluble components, serving as an indicator of overall quality. Heavy metal limits (Pb, Cd, As, Hg, Cu) ensure safety compliance with international standards.

## Data Availability

No new data were created or analyzed in this study. Data sharing is not applicable to this article.

## Abbreviations

The following abbreviations are used in this manuscript:

AAP	acidic *Angelica* polysaccharide
AASP	alkaline soluble polysaccharide
ACD	anemia of chronic disease
ADM	adriamycin
ALI	acute lung injury
Akt	protein kinase B
AP-1	activator protein-1
ASP/APS	*Angelica sinensis* Polysaccharides
A-SFE	*Angelica sinensis* supercritical extract
BALF	bronchoalveolar lavage fluid
Bax	Bcl-2 associated X protein
Bcl-2	B cell lymphoma/leukemia-2
BMMs	bone marrow macrophage(s)
BMSCs	bone marrow-derived mesenchymal stem cells
Breg	regulatory B cell
c-Jun	c-Jun N-terminal kinase
CD3^+^	cluster of differentiation 3
CD4^+^ T cell	helper T cell
CD4SP	CD4^+^ single positive T lymphocytes
CD8^+^	cytotoxic T lymphocyte
CFA	complete Freund’s adjuvant
Con A	concanavalin A
COX-2	cyclooxygenase-2
CRC	colorectal cancer
DC-STAMP	dendritic cells-specific transmembrane protein
DC2.4	murine dendritic cell line DC2.4
DMT1	divalent metal ion transporter-1
EPO	erythropoietin
ERK	extracellular signal-regulated kinase
FLS	fibroblast-like synoviocytes
FoxO1	forkhead box protein O1
Foxp3	forkhead box protein p3
GC-MS	Gas Chromatography–Mass Spectrometry
G-CSF	granulocyte colony-stimulating factor
G-M	granulocyte macrophage
Gab2	GRB2-associated binding protein 2
GMCs	glomerular mesangial cells
HCT	hematocrit
HIF2α	Hypoxia-Inducible Factor 2α
HMGB1	high mobility group box 1
HSC	hepatic stellate cell
HPGPC	High-Performance Gel Permeation Chromatography
HPLC	High-Performance Liquid Chromatography
IBD	inflammatory bowel disease
IFN-γ	interferon-γ
Ig	immunoglobulin
IgE	immunoglobulin E
IPEC-J2	intestinal porcine epithelial cell-J2
IKKs	iκB kinases
IL	interleukin
IL-6R	interleukin-6 receptor
iNOS	inducible nitric oxide synthase
IR	insulin resistance
IRF-3	interferon regulatory factor 3
iROS	intracellular reactive oxygen species
IκB	Inhibitor of NF-κB
JAK2	janus kinase 2
LIGc	ligusticum cycloprolactam
LPS	Lipopolysaccharide
LSZ	lysozyme
M-CSF	macrophage colony-stimulating factor
MAP	mitogen-activated protein
MAPK	mitogen-activated protein kinase
MDSC	myeloid-derived suppressor cell
MHC	major histocompatibility complex
MIP-2	macrophage inflammatory protein-2
MMP	matrix metalloproteinase
MN	membranous nephropathy
MPO	myeloperoxidase
MS	Mass Spectrometry
MyD88	myeloid differentiation primary response gene 88
NE	neutrophilic granulocyte
NF-κB	nuclear factor kappa-B
NFATc1	nuclear factor of activated T cells c-1
NK cell	natural killer cell
NLRP3	NOD-like receptor family pyrin domain containing 3
NMR	Nuclear Magnetic Resonance
NO	nitric oxide
Nfr2	nuclear factor erythroid 2-related factor 2
OA	osteoarthritis
OC	osteoclast
p-SMAD	phosphorylated SMAD
p-STAT3	phosphorylated signal transducer and activator of transcription 3
PGE_2_	prostaglandin E 2
PI3K	phosphoinositide 3 kinase
PLGA	polylactic acid glycolic acid copolymer
PPARγ	peroxisome proliferator-activated receptor γ
RA	rheumatoid arthritis
RANKL	receptor activator for nuclear factor-κB ligand
RCC	renal cell carcinoma
ROS	reactive oxygen species
RUNX2	recombinant runt related transcription factor 2
RXFP1	relaxin receptor 1
SCI	spinal cord injury
Sirt1	sirtuin 1
SMAD	sma- and mad-related proteins
SOX-9	sex-determining region Y-box 9
STAT3	signal transducer and activator of transcription 3
STAT5	signal transducer and activator of transcription 5
T cell	T lymphocyte
TAMs	tumor-associated macrophages
TECs	thymic epithelial cells
TfR1	transferrin receptor 1
TGF-β	transforming growth factor-β
Th1	T helper 1 cell
Th17	T helper 17 cell
Th2	T helper 2 cell
TLR4	toll-like receptor 4
TNF-α	tumor necrosis factor-alpha
Treg cell	regulatory T cell
TRIF	toll/IL-1 receptor homologous region
VEGF	vascular endothelial growth factor
Z-LIG	(Z)-ligustilide
